# A synthetic genomics-based African swine fever virus engineering platform

**DOI:** 10.1126/sciadv.adu7670

**Published:** 2025-03-26

**Authors:** Walter Fuchs, Nacyra Assad-Garcia, Hussein M. Abkallo, Yong Xue, Lauren M. Oldfield, Nadia Fedorova, Alexandra Hübner, Tonny Kabuuka, Katrin Pannhorst, Dirk Höper, Vishvanath Nene, Norberto Gonzalez-Juarbe, Lucilla Steinaa, Sanjay Vashee

**Affiliations:** ^1^ Institute of Molecular Virology and Cell Biology, Friedrich-Loeffler-Institut, Federal Research Institute for Animal Health, 17493 Greifswald-Insel Riems, Germany.; ^2^ J. Craig Venter Institute, Rockville, MD 20850, USA.; ^3^ International Livestock Research Institute (ILRI), Nairobi 00100, Kenya.; ^4^ National Agricultural Research Organization (NARO), P.O. Box 295, Entebbe, Uganda.; ^5^ Institute of Diagnostic Virology, Friedrich-Loeffler-Institut, Federal Research Institute for Animal Health, 17493 Greifswald-Insel Riems, Germany.

## Abstract

African swine fever (ASF) is a deadly viral disease in domestic pigs that has a large global economic impact for the swine industry. It is present in Africa, Europe, Asia, and in the Caribbean island of Hispaniola. There are no effective treatments or broadly licensed vaccines to prevent disease. Efforts to counteract ASF have been hampered because of the lack of convenient tools to engineer its etiological agent, ASF virus (ASFV), largely due to its large noninfectious genome. Here, we report the use of synthetic genomics methodology to develop a reverse genetics system for ASFV using a CRISPR-Cas9–inhibited self-helper virus to reconstitute live recombinant ASFV from synthetic genomes to rapidly generate a variety of combinatorial mutants of ASFV. The method will substantially facilitate the development of therapeutics or subunit and live-attenuated vaccines for ASF. This synthetic genomics-based approach has wide-ranging impact because it can be applied to rapidly develop reverse genetics tools for emerging viruses with noninfectious genomes.

## Teaser

A rapid ASFV engineering method was developed to facilitate the generation of countermeasures for ASF, a deadly pig disease.

## INTRODUCTION

African swine fever (ASF) is a devastating disease that affects pigs, wild boars, and feral pigs, manifesting as a hemorrhagic disease with severe damage to the organs and with up to 100% mortality rate. ASF was first described in Kenya in 1921 (*1*) and spread to western European countries in the mid-20th century, before being eradicated in 1995 via euthanasia of farm herds and hunting of wild carriers, with the exception of Sardinia (*2*). However, in 2007, ASF was introduced into Georgia by transport of contaminated meat products (*3*) and then expanded further to central and eastern Europe (*4*). More recently, since 2018, ASF has been reported in many parts of Asia, from China down to Papua New Guinea, where it caused great economic losses since Asia accounts for approximately 50% of the global pig population (*5*). Furthermore, ASF is now present in about 26 countries in sub-Saharan Africa, where the virus is enzootic with regular epizootic outbreaks in many countries. Also alarming is the news that ASF has now spread to the Western Hemisphere with the confirmation of at least 24 outbreaks in the Dominican Republic and several in Haiti since 2021 (*6*, *7*). Currently, in most countries, the only control options for ASF are culling and biosecurity measures (*8*). Since 2022, live-attenuated ASFV-G-ΔI177L and ASFV-G-ΔMGF have been tested and shown efficacy against parental virus infection in Vietnam (*9*, *10*). As a result, vaccines based on these strains (NAVET-ASFVAC and AVAC ASF LIVE, respectively) have been licensed in Vietnam. However, a very recent study challenging NAVET-ASFVAC or AVAC ASF LIVE–vaccinated pigs with a genotype I/II strain found that they were not completely protective (*11*), and so, their general applicability in ASF control remains to be demonstrated.

ASF is caused by a double-stranded DNA (dsDNA) virus in the family *Asfarviridae*, sharing similarities with other large dsDNA viruses, such as the poxviruses and iridoviruses (*12*). The viral genome from different isolates can vary in size from 170 to 194 kilobase pairs (kbps) and contains up to 167 conserved genes (*8*). However, more recent studies suggested that ASF virus (ASFV) contains even higher numbers of up to 235 protein-encoding open reading frames (ORFs), depending on the genotype (*13*). To date, 23 different genotypes of ASFV based on the major capsid protein (p72) gene sequence have been identified in Africa (*14*, *15*). Geographic locations are associated with distinct clades in the phylogenetic tree. For example, genotype IX and X, which are very similar, appear to be the most predominant genotypes in East Africa and have been associated with disease outbreaks (*15*). Various genotypes have also been associated with disease in West (I), Central (I, IX, and X) and Southern Africa (multiple, including II, VIII, XIII, and XXII) (*15*). In contrast, the current panzootic ASF outside Africa is mainly caused by genotype II (*5*). However, a reemergence of genotype I virus in China has been reported (*16*) as well as the emergence of a virulent genotype I/II recombinant virus in China, Vietnam, and Russia (*11*, *17*, *18*).

Currently, there are multiple research groups that are actively trying to develop a safe and effective vaccine against ASF using different approaches. One approach in ASF vaccine development has been to use the classical approach of strains attenuated by serial passaging. It has been shown that adaptation to cell line culturing is associated with increasing number of deletions of genes in the ends of the viral genome in some cell lines (*19*). Unfortunately, immunization with these cell line–adapted ASFV strains has, thus far, not induced full protection against the parental strain (*19*, *20*). Another approach to creating live-attenuated vaccines involves targeted deletion of genes from virulent wild-type ASFV (*9*, *21*). This strategy has been hampered by several constraints which include the time and substantial effort required to generate and purify recombinant viruses from plasmid-transfected cells infected with wild-type viruses. In particular, the introduction of simultaneous multiple gene deletions has proved to be difficult. Although the Cre/LoxP-based recombination system for gene deletions allows the reuse of selection markers, the individual deletions have to be performed sequentially (*22*). The recent use of the CRISPR-Cas9 system has greatly improved recombination efficiency [as reviewed by Zhang (*23*)], but the main time-consuming task remains the isolation of a mutant virus from the parental wild-type virus. This task requires many successive rounds of plaque/foci picking, which can, depending on the host cells used, change the virus in undesired ways and thus, complicates the isolation of desired mutants. Thus, there is a need to apply innovative and more efficient technologies, such as synthetic biology/genomics, to identify molecular markers of pathogenesis in the ASFV genome and rationally generate attenuated strains that may serve as vaccines.

Advances in synthetic biology and synthetic genomics provide easy and efficient tools to engineer large DNA molecules, including virus genomes (*24*). Furthermore, methods based on CRISPR-Cas technology have revolutionized the efficient manipulation of cellular genomes and large DNA viruses, such as herpesviruses (*25*, *26*) and vaccinia virus (*27*). Recently, this genome editing methodology has been expanded to ASFV (*28*). In addition, methods have been developed that enable the in vivo (*29*) and in vitro (*30*) assembly of large DNA fragments, ranging from hundred kilobases to even whole megabase bacterial genomes, as was demonstrated by the J. Craig Venter Institute (JCVI) in creating a bacterial cell controlled by a chemically synthesized genome (*31*). The in vivo DNA assembly methods, based on transformation-associated recombination (TAR), have been extended to engineer human herpesviruses (*32*, *33*). Together, these methods can be applied to not only establish a vaccine platform for transboundary animal pathogens, such as ASFV, but also facilitate the study of their biology and pathogenesis. Essentially, the synthetic biology/genomics reverse genetics approach allows a workflow conducive to a high-throughput production of a variety of modified ASFV genomes/viruses for functional studies and that can ultimately generate vaccine candidates.

**Fig. 1.**
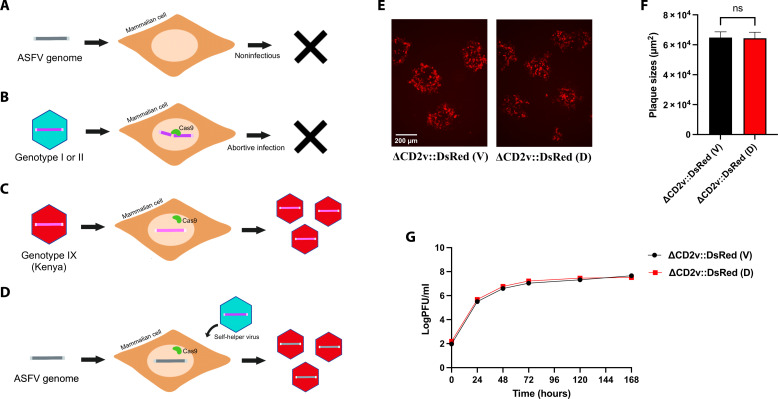
Reconstitution of live ASFV from transfected genomic DNA using a self-helper virus. (**A**) The ASFV genome is by itself not infectious since transfection of the viral genome into mammalian cells does not produce virus progeny. (**B**) Replication of ASFV genotype I or II is inhibited in mammalian cells when the p30 gene is targeted for cleavage by a gRNA-directed Cas9 nuclease. (**C**) Replication of ASFV genotype IX is not inhibited in mammalian cells when the p30 gene is targeted by the same gRNA-directed Cas9 nuclease and produces virus progeny. This is due to single-nucleotide polymorphisms at the target site between the different genotypes. (**D**) Hypothesis: An ASFV genome can be booted-up by a self-helper virus (either a heterologous or homologous ASFV) whose replication is inhibited by CRISPR-Cas9 cleavage activity as long as the donor genome is immune to the same cleavage activity. (**E**) Fluorescence images of representative plaques produced by the reconstituted virus [ΔCD2v::DsRed (D)] and the parental virus [ΔCD2v::DsRed (V)] at 6 days postinfection of WSL cells. ns, not significant. (**F**) Plaque areas (square millimeter) produced by the same viruses. They were measured 6 days after infection of WSL cells. Shown are the mean sizes of 30 plaques per virus with positive standard deviations. (**G**) Replication of the reconstituted virus [ΔCD2v::DsRed (D)] on WSL cells. Progeny virus titers [plaque-forming unit (PFU) per milliliter] of ΔCD2v::DsRed (D) and parental virus [ΔCD2v::DsRed (V)] were determined at the indicated times after infection at an MOI of 0.03. Shown are the mean results of at least three parallel experiments.

A major barrier to establishing a reverse genetics system for ASFV is that its genome is not infectious (Fig. 1A) unlike those of other viruses, such as human herpesviruses and positive-strand RNA viruses. Since ASFV mainly replicates in the host cell cytoplasm, its dsDNA genome requires its own (viral) DNA polymerase and accessory proteins for initiating replication. In addition, ASFV transcription uses its own virus-encoded RNA polymerase, transcription factors, and mRNA-processing enzymes, all of which are present in the virus particle and required to initiate replication (*8*). For viruses whose genomes are not infectious, support plasmids that encode viral polymerases and accessory proteins are necessary to help reconstitute virus from recombinant genomes. As an example, the respiratory syncytial virus (RSV) recombinant genomes can be reconstituted in host cells by expressing the RSV *N*, *P*, *L*, and *M2-1* genes—genes that encode for viral proteins that constitute the viral polymerase complex (*34*). Given that ASFV has more than 150 genes (*8*), compared to 11 for RSV (*34*), it has been difficult to identify the minimal and key helper genes necessary to reconstitute ASFV from recombinant DNA. As an alternative, helper viruses have been used to reconstitute other viruses whose genomes are not infectious. For example, attenuated fowlpox virus has been used to reconstitute vaccinia virus from recombinant genomes, while live horsepox virus was reconstituted from recombinant overlapping DNA fragments by recombination in cells infected with Shope fibroma virus (*35*). However, to date, no helper genes or heterologous viruses have been identified to reconstitute live virus from purified ASFV DNA.

Here, we report the use of a self-helper virus to develop a synthetic genomics-based reverse genetics method for ASFV. Using a genotype IX ASFV strain, Kenya-IX-1033 (*36*), as a proof of principle for the reverse genetics method, we generated various recombinant ASFV mutant strains that contained single or combinations of modifications across the ASFV genome and strains that contain a fluorescent protein fusion of either CD2v or K145R. The establishment of this innovative reverse genetics method should facilitate the development of safe and effective vaccines against ASF as well as the study of pathogenesis and biology of ASFV. The use of a built-in self-helper virus together with a synthetic genomics genome assembly system should have wide impact to rapidly develop reverse genetics for viruses with noninfectious genomes, especially new or emerging viruses of pandemic concern.

## RESULTS

### Use of a self-helper virus to reconstitute live virus from ASFV-Kenya-IX-1033 virion DNA

It has previously been reported that replication of recombinant genotype I ASFV-BA71VΔTKDsRed and genotype II ASFV-Armenia-2008 viruses were efficiently inhibited in an ASFV-permissive wild boar lung cell line (WSL) that expresses Cas9 and a guide RNA (gRNA) targeting the essential ASFV p30 gene (WSL-gRp30) (Fig. 1B) (*37*). In contrast, replication of a genotype IX mutant, ASFV-Kenya-IX-1033, ΔCD2v::DsRed (ΔCD2v::DsRed) (*37*), which expresses red fluorescent protein, was not affected at all in WSL-gRp30 (Fig. 1C) due to a 4–base pair (bp) difference in the *CP204L* sequence between either ASFV-BA71V or ASFV-Armenia and ASFV-Kenya-IX-1033 (*37*) that is targeted by the gRNA. Given these results, we hypothesized that an ASFV strain whose replication is efficiently inhibited by a mechanism, such as Cas9 cleavage in host cells, should serve as a self-helper virus to package or “boot up” virion DNA of an ASFV strain that is immune to the inhibiting mechanism for virus reconstitution (Fig. 1D). This idea is similar to the use of conditionally lethal “helper” bacteriophages to transfect isolated wild-type DNA in the 1960s and early 1970s (*38*). Thus, to test this hypothesis, we selected two recombinant ASFV strains, ASFV-ArmeniaΔ285L::GFPhuCD4 (*39*) and ASFV-NHVΔTK::GFP (*40*), both of which express green fluorescent protein (GFP) and are inhibited by Cas9 cleavage of the p30 gene, as the self-helper viruses and ΔCD2v::DsRed as the donor genome for virus reconstitution. After transfection of the ΔCD2v::DsRed genome into normal WSL cells and subsequent infection with either ASFV-ArmeniaΔ285L::GFPhuCD4 or ASFV-NHVΔTK::GFP, mainly green-fluorescent, but only a few red- or dual-fluorescent foci were observed. In contrast, when tested for virus reconstitution in WSL-gRP30 cells under the same conditions, mainly red-fluorescent foci were detected and could be easily purified to homogeneity in subsequent plaque assays, indicating successful reconstitution of the transfected ΔCD2v::DsRed genome.

To test for any potential recombination events between the transfected ΔCD2v::DsRed genome and the self-helper virus, DNA from 10 red progeny plaque isolates, from either ASFV-ArmeniaΔ285L::GFPhuCD4 or ASFV-NHVΔTK::GFP self-helper virus infections of either normal WSL or WSL-gRp30 cells, were used as template to amplify and sequence five approximately 500-bp fragments from different regions across the ASFV genome. Results from these experiments showed that 49 of the 50 fragments originated from ASFV-Kenya-IX-1033, whereas only one originated from the ASFV-NHVΔTKG self-helper virus (table S1). The single helper virus sequence in that isolate was located at the essential p30 locus, but which was not targeted by Cas9, as this particular reconstitution experiment was performed on normal WSL cells (fig. S1 and table S1). These results suggest that recombination between the transfected ASFV DNA and the ASFV helper viruses, ASFV NHV (GenBank, #KM262845, DNA sequence identity approx. 79%) or ASFV Armenia 2008 (sequence identity approx. 85%) is limited likely because of less homology. Whole-genome sequencing of one of the reconstituted virus isolates [no. 7 from table S1; henceforth ΔCD2v::DsRed (D)] showed that it was completely (>99.9%) identical to ΔCD2v::DsRed (GenBank, #PQ323358) and contained no ASFV-NHV–derived sequences, confirming that live virus was reconstituted from the transfected ΔCD2v::DsRed genome (table S2).

The reconstituted ΔCD2v::DsRed (D) was characterized using plaque size and growth kinetics assays on WSL cells. As expected, it exhibited similar plaque sizes as the parental ΔCD2v::DsRed (V) (Fig. 1, E and F). In addition, ΔCD2v::DsRed (D) replicated with almost identical kinetics as parental ΔCD2v::DsRed (V) (Fig. 1G). Together, these results validate our hypothesis that a self-helper virus can be used to reconstitute virus from a transfected noninfectious genome.

### A synthetic genomics reverse genetics system for ASFV-Kenya-IX-1033 using a heterologous ASFV helper virus

**Fig. 2.**
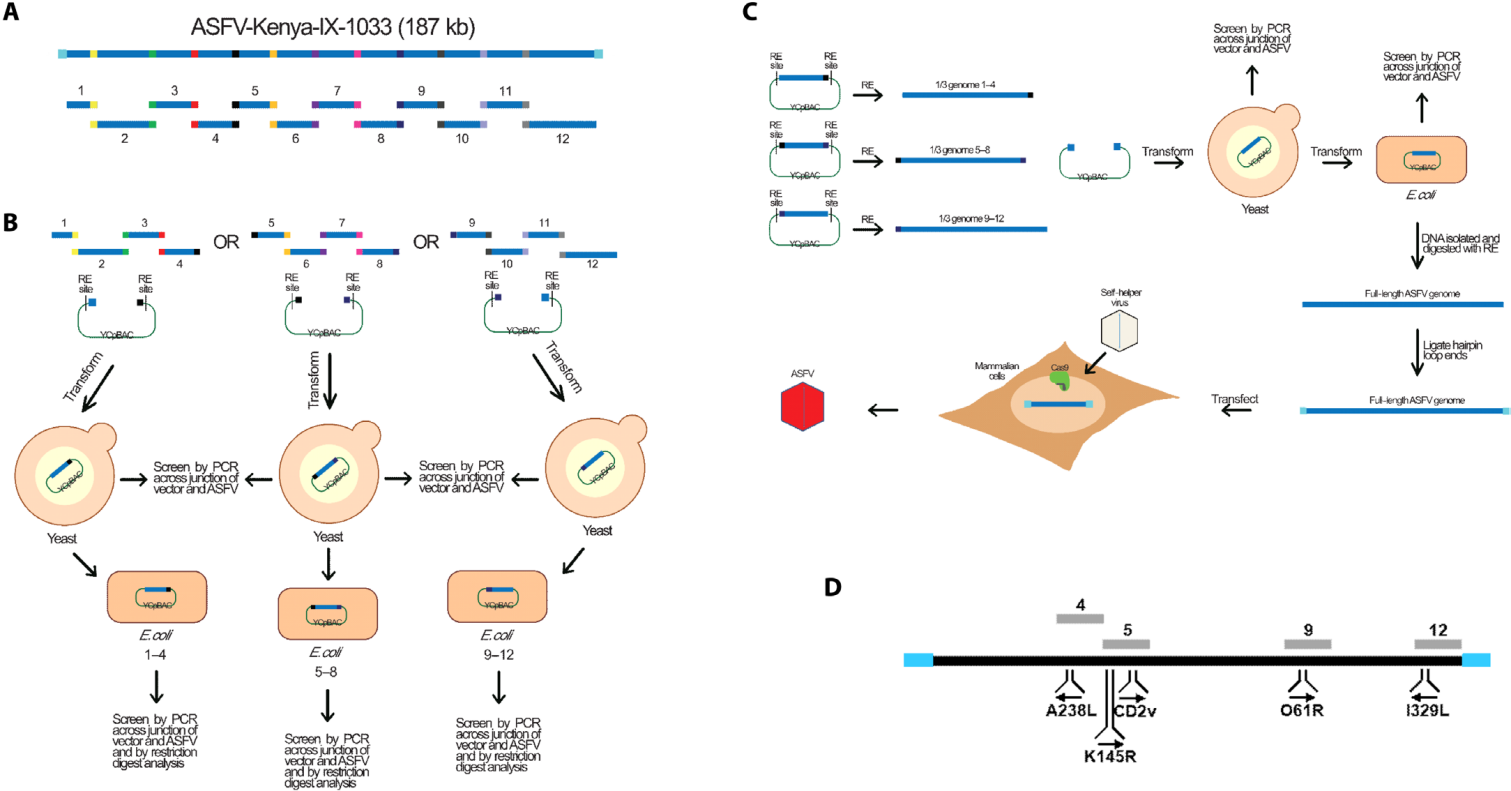
Schematic depiction of the synthetic genomics assembly of the ASFV-Kenya-IX-1033 genome. (**A**) Deconstruction of ASFV-Kenya genome. Twelve approximately 15-kb overlapping genomic fragments span the entire sequence of the ASFV-Kenya genome. (**B**) TAR-assembly of third genomes. Third genomes of ASFV-Kenya were assembled separately from fragments 1 to 4, fragments 5 to 8, and fragments 9 to 12. The corresponding fragments were pooled with a linear YCpBAC vector that contained terminal homology to each end of the pertinent third genome and flanked by a unique restriction enzyme (RE) site and then transformed into yeast cells. Transformants were screened by PCR amplification for correct assembly. DNA from the positive transformants was isolated and transformed into *E. coli* cells to produce high concentration DNA stocks. (**C**) TAR assembly of full-length genome and reconstitution of live recombinant virus. Plasmid DNAs of the three third genomes were isolated and digested to release the ASFV-Kenya fragments. The third fragments were transformed into yeast cells together with a linear YCpBAC vector that contained homology to the terminal repeats of ASFV flanked by unique restriction sites and a different marker for selection in yeast that was used for the third genome assemblies. DNA from the positive transformants was isolated and transformed into *E. coli*. DNA was isolated from *E. coli*, and the viral genome was released by digestion with an RE targeting the flanking sites. Hairpin loops were ligated to the viral genome and transfected into WSL cells that contained the CRISPR-Cas9 system. The transfected cells were then infected by a self-helper virus to reconstitute live recombinant ASFV-Kenya. (**D**) Schematic of genes targeted for modification. ASFV genes that were modified are shown. The numbered gray segments represent the fragments containing the targeted genes. The blue rectangles represent the terminal hairpin loops at the ends of the ASFV genome.

With the successful development of the self-helper virus boot up method for virus reconstitution, we then established a synthetic genomics genome assembly process to assemble ASFV genomes, analogous to one that we previously reported for human herpesviruses (Fig. 2) (*32*, *33*). We assembled full-length ASFV Kenya-IX-1033 “synthetic” genome by TAR in yeast from 12 overlapping wild-type or modified fragments or “parts” (Fig. 2A and table S3) (see the Supplementary Materials for detailed results). However, because of the repeat sequences at the genomic ends, the genomes were assembled in two steps, first as three individual overlapping one-third genomes (1–4, 5–8, and 9–1*2*) from TAR-cloned fragments (Fig. 2B) and then as full-length genomes from the one-third genomes (Fig. 2C). At each step, the assemblies were transformed into *Escherichia coli* for isolation of higher amounts of DNA after appropriate characterization by junction polymerase chain reaction (PCR) amplification (figs. S2 and S3).

**Fig. 3.**
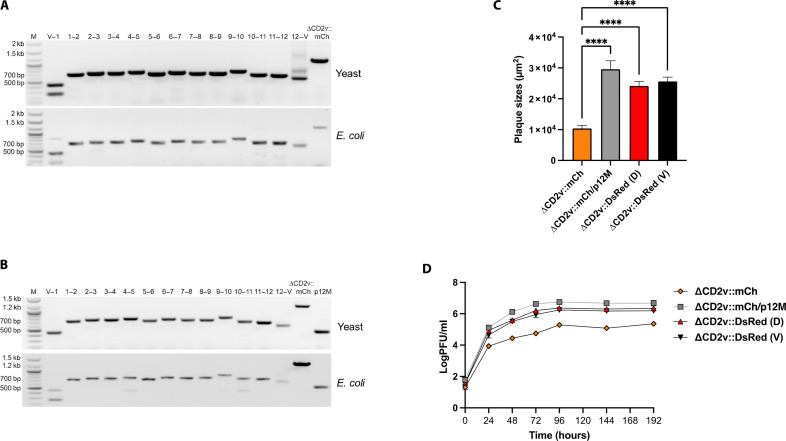
Characterization of the TAR-assembled synthetic ASFV-Kenya viruses. (**A**) Junction PCR amplification analysis of assembled full-length DCD2v::mCh genome. Representative agarose gel after PCR amplification showing the presence of all the appropriate junctions between the ASFV-Kenya fragments (1, 2, etc.). For the lane headings, numbers give the ASFV-Kenya fragment numbers and V refers to vector for the junctions tested. The positions of the molecular size markers in the M lane are given to the left of the gel. (**B**) Junction PCR amplification analysis of assembled full-length ΔCD2v::mCh/p12M genome. Representative agarose gel after PCR amplification showing the presence of all the appropriate junctions between the ASFV-Kenya fragments (1, 2, etc.). For the lane headings, numbers give the ASFV-Kenya fragment numbers and V refers to vector for the junctions tested. PCR amplification of the mutated segment of the p12 gene is also shown in the rightmost lane. The positions of the molecular size markers in the M lane are given to the left of the gel. (**C**) Plaque areas (square millimeter) produced by the TAR-assembled synthetic viruses. They were measured 6 days after infection of WSL cells and compared to booted-up ΔCD2v::DsRed (D) and parental ΔCD2v::DsRed (V) viruses. Shown are the mean sizes of >50 plaques per virus with positive standard deviations. For non-parametric multiple groups analyses, we used a Kruskal-Wallis H test with Dunn’s post hoc analysis. Asterisks denote the level of significance observed: ****P ≤ 0.0001. (**D**) Replication of the reconstituted synthetic viruses on WSL cells. Progeny virus titers (plaque-forming unit per milliliter) of ΔCD2v::mCh, ΔCD2v::mCh/p12M, ΔCD2v::DsRed (D) and ΔCD2v::DsRed (V) were determined at the indicated times after infection at an MOI of 0.03. Shown are the mean results of at least three parallel experiments.

To complete the reverse genetics system for ASFV, we sought to reconstitute live virus from the assembled synthetic ASFV genomes using the self-helper virus (see above). Given that it was demonstrated using the genome of a red fluorescent ASFV CD2v mutant, an analogous full-length synthetic genome that contained the replacement of the entire *CD2v* gene by mCherry (ΔCD2v::mCh) was assembled. This was accomplished by first modifying fragment 5 (Fig. 2D) with the desired changes using CRISPR-Cas9 modification in vitro and TAR assembly in yeast (fig. S4A), identifying the modified clones by PCR amplification and then transforming the correct clone into *E. coli* (fig. S4, A and B), followed by assembling the full-length genome from the modified fragment 5 and the remaining wild-type parts in two steps (Fig. 3A). To reconstitute live virus, the full-length synthetic ΔCD2v::mCh genome was released from the YCpBAC by I-SceI digestion and, since native ASFV genomes have hairpin loops at the ends, ligated it to synthetic hairpin loop–forming oligonucleotides (Fig. 2). Their sequences were either adopted from the published ones of ASFV strain BA71V (*41*) or deduced from our sequencing of the ASFV-Kenya-IX-1033 genome (table S6) (*36*). The hairpin loop–containing synthetic genome was transfected into WSL-gRp30 cells, which were subsequently infected with genotype II helper virus ASFV-ArmeniaΔ285L::GFPhuCD4. Unexpectedly, considerable proportions of the virus progenies formed green or double-fluorescent plaques, suggesting that there was recombination between the transfected DNA and the helper virus genome (fig. S1). However, numerous progeny viruses also exhibited the desired single red fluorescence.

To verify whether these progeny viruses were indeed reconstituted from the assembled DNA, several clones were plaque purified, from which genomic DNA was isolated and then analyzed by PCR amplification and Sanger sequencing of the *CP240L* (p30) gene region and, partly, other loci (*K146R* and *E199L*), which all exhibited the wild-type Kenya-IX-1033 sequence. However, helper virus sequences were frequently detected within the terminal inverted repeats. The genomes of three ΔCD2v::mCh isolates were sequenced. “Heterologous” helper virus–derived sequences were found at one genome end in two of them, whereas, no helper virus sequences were detected in the third ΔCD2v::mCh isolate (fig. S1 and table S2), demonstrating that virus reconstitution from synthetic assembled ASFV genomes is possible. However, these synthetic ΔCD2v::mCh isolates, including the one that contained only the Kenya-IX-1033 sequence exhibited smaller plaque sizes (Fig. 3C, green) and grew at significantly lower titers on WSL cells (Fig. 3D, green), compared to the conventionally generated [ΔCD2v::DsRed (V)] and reconstituted [ΔCD2v::DsRed (D)] mutants (Fig. 3, C and D, black and red, respectively). These results suggest that the artificial genome ends of the synthetic constructs, consisting of partial I-SceI restriction sites and ligated hairpin-forming oligonucleotides are partially, but not fully, functional.

### Improved synthetic genomics reverse genetics system using homologous ASFV-Kenya-IX-1033 as helper virus

To overcome the issues posed by the artificial genome ends, we attempted to use the homologous Kenya-IX-1033 virus itself as the helper virus to reconstitute virus from the synthetic assembled genomes, which would allow repair of the defective artificial ends by homologous recombination. We previously observed that replication of all hitherto tested ASFV strains, including Kenya-IX-1033, was efficiently inhibited in a WSL cell line that expressed Cas9 and a gRNA targeting the essential p12 gene (WSL-gRO61R). Thus, we postulated that a synthetic assembled Kenya-IX-1033 genome containing a Cas9-resistant–modified but functional p12 gene should be reconstituted by the homologous helper virus in the WSL-gRO61R cell line and have wild-type growth characteristics due to recombination at the genome termini to restore the proper sequence and structure needed for replication. To test this postulate, the p12 gene (*O61R*, fragment 9; Fig. 2D) was modified by introducing silent mutations at the gRNA targeting locus such that it would not be cleaved by Cas9 while simultaneously preserving the proper amino acid sequence (fig. S5A). A full-length genome that contained the p12 modification as well as the mCherry at the *CD2v* locus (ΔCD2v::mCh/p12M) was assembled from the modified parts, fragments 5 and 9, and the remaining wild-type parts. The genome was confirmed for full-length assembly by junction PCR amplification in yeast and after subsequent transfer to *E. coli* (Fig. 3B). After digestion with I-SceI and ligation with hairpins, the assembled genome was transfected into WSL-gRO61R cells, which were then infected with a derivative mutant of Kenya-IX-1033, ASFV-Kenya-IX-1033ΔDUT::GFPhuCD4 (ΔDUT::GFP), a dUTPase-negative mutant that contains a wild-type p12 gene susceptible to Cas9 cleavage. Green-, red-, and double-fluorescent plaques were obtained. Analysis of the resulting virus progenies from the green- and double-fluorescent plaques revealed that recombination had occurred between the genomes of the donor and homologous helper virus since they contained the p12 modification and for the double-fluorescent viruses, the ΔCD2v::mCh modification as well (figs. S1 and S6). Specifically, 87% of the total fluorescent plaques were helper virus that only acquired the p12 Cas9-resistant modification, and 8% were recombinant viruses that contained both the expected ΔCD2v::mCh modification and the ΔDUT::GFP modification from the helper virus (fig. S6). However, all of the desired red-fluorescent plaques (5% of the total fluorescent plaques; fig. S6) tested contained the Cas9-resistant p12 gene modification and the desired CD2v deletion. The ΔCD2v::mCh/p12M isolates (Fig. 3, C and D, blue) exhibited similar plaque sizes and grew with similar, if not slightly better, growth kinetics compared to the conventionally generated [ΔCD2v::DsRed (V)] or reconstituted [ΔCD2v::DsRed (D)] mutants (Fig. 3, C and D, black and red, respectively), suggesting that the genomes of the synthetic recombinant viruses contained the correct sequence at the termini, likely by recombination of the ends of the homologous helper virus genome and the synthetic assembled genome.

### Combinatorial and genome-wide engineering of ASFV-Kenya-IX-1033

With the successful reconstitution of virus with wild-type–like growth characteristics from the synthetic assembled genome, we then sought to demonstrate the utility of this synthetic genomics reverse genetics system to engineer ASFV in a combinatorial, genome-wide manner. To do this, we targeted the *A238L*, *K145R*, and *I329L* genes for deletion individually and in combination since each of these genes has been reported to be nonessential individually for viral replication in other ASFV backgrounds (*39*, *42*–*44*). The *A238L*, *K145R*, and *I329L* genes reside in fragments 4, 5 and 12, respectively, in our system (Fig. 2D). Thus, these fragments were individually modified using in vitro CRISPR-Cas9 editing to replace *K145R* with mCherry and to delete *A238L* and *I329L* (fig. S5, B to D, respectively). The modified fragments were then assembled with the other wild-type parts in a mix-and-match manner to generate various full-length genomes that contained single, double, and triple deletions in the Cas9-resistant p12 mutant background. As before, each of these genomes was confirmed for full-length assembly in both yeast and *E. coli* by junction PCR amplification (fig. S7).

**Fig. 4.**
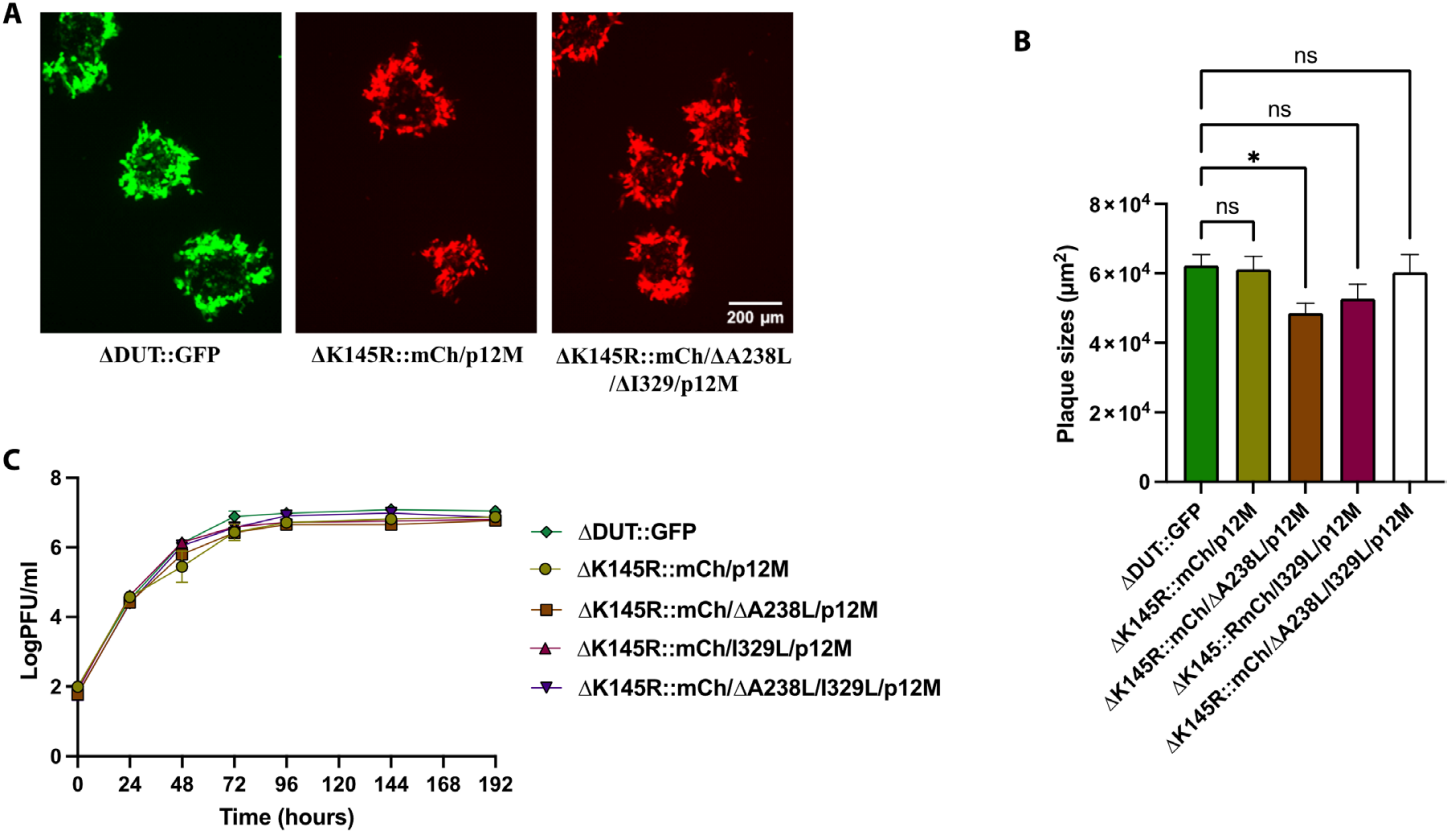
Characterization of combinatorial ASFV-Kenya mutant viruses. (**A**) Fluorescence images of representative plaques produced by the viruses reconstituted from synthetic assembled genomes, ΔK145R::mCh/p12M and ΔK145R::mCh/ΔA238L/ΔI329L/p12M, compared to the self-helper virus, ΔDUT::GFP, at 6 days postinfection of WSL cells. (**B**) Plaque areas (square millimeter) produced by the analyzed viruses. They were measured 6 days after infection of WSL cells and compared to ΔDUT::GFP. Shown are the mean sizes of >50 plaques per virus with positive standard deviations. For non-parametric multiple groups analyses, we used a Kruskal-Wallis H test with Dunn’s post hoc analysis. Asterisks denote the level of significance observed: *P ≤ 0.05. (**C**) Replication of the reconstituted viruses on WSL cells. Progeny virus titers (plaque-forming unit per milliliter) of ΔK145R::mCh/p12M, ΔK145R::mCh/ΔA238L/p12M, ΔK145R::mCh/ΔI329L/p12M, ΔK145R::mCh/ΔA238L/ΔI329L/p12M, and control virus ΔDUT::GFP were determined at the indicated times after infection at an MOI of 0.03. Shown are the mean results of at least three parallel experiments.

The genomes were processed and transfected as before into WSL-gRO61R cells, which were then infected with wild-type Kenya-IX-1033 or ΔDUT::GFP to reconstitute the synthetic recombinant viruses. Although the resulting red, green, dual-fluorescent, and nonfluorescent virus plaques were observed in transfections for each mutant, only the red virus plaques were propagated and tested by PCR amplification for the expected changes. In all, we obtained live virus for ΔK145R::mCh/p12M, ΔK145R::mCh/ΔA238L/p12M, ΔK145R::mCh/ΔI329L/p12M, and ΔK145R::mCh/ΔA238L/ΔI329L/p12M. All analyzed viruses contained the silent mutations introduced in the p12 gene of the synthetic genomes, demonstrating the efficiency of the CRISPR-Cas9–based selection. However, not every red virus progeny contained each of the expected deletions of *A238L* and/or *I329L*, suggesting that multiple recombination events occurred between the transfected synthetic assembled genomes and the helper virus genome (fig. S1). For example, of the two triple deletion mutant progeny analyzed, one contained the expected three deletions, whereas the other contained two deletions (ΔK145R::mCh and ΔA238L) but a wild-type *I329L* gene. The viruses were characterized by plaque sizes and growth kinetics after infection of WSL cells at a multiplicity of infection (MOI) of 0.03 (Fig. 4). The single mutant, ΔK145R::mCh/p12M had similar plaque sizes and grew at essentially the same rate as the homologous helper virus, ΔDUT::GFP (Fig. 4, A to C). The double mutants, ΔK145R::mCh/ΔA238L/p12M and ΔK145R::mCh/ΔI329L/p12M, as well as the triple mutant, ΔK145R::mCh/ΔA238L/ΔI329L/p12M, grew similarly as the ΔDUT::GFP self-helper virus and the single mutant, ΔK145R::mCh/p12M (Fig. 4C). However, the ΔK145R::mCh/ΔA238L/p12M double mutant gave rise to smaller plaques than all of the other viruses (Fig. 4B), but it remains to be determined whether this phenotype may be due to mutations at untargeted genome positions. The assembled genome isolated from *E. coli* and resulting viral genome of the triple mutant, ΔK145R::mCh/ΔA238L/ΔI329L/p12M were sequenced (table S7). Comparison of the sequences revealed that the genome hairpin loop ends were restored in the recombinant virus, presumably by recombination with the homologous helper virus. In addition, there were differences in the lengths of four noncoding G or C homopolymers as well as four single nucleotide differences, with two of them leading to deduced amino acid substitutions in *MGF 505-2R* and *D250R*, respectively (table S7). It is not known whether these minor mutations resulted from recombination with the helper virus or during plaque purification and propagation. Of importance, the mutations do not appear to affect plaque size and growth kinetics of ΔK145R::mCh/ΔA238L/ΔI329L/p12M since they were very similar to the single mutant, ΔK145R::mCh/p12M and the ΔDUT::GFP self-helper virus (Fig. 4, B and C).

To further demonstrate the utility of the ASFV synthetic genomics reverse genetics system, recombinant viruses containing C-terminal fusions of mCherry with CD2v (CD2v-mCh/p12M) or Cerulean with K145R (K145R-Cer/p12M) were generated since they can be used for live-cell imaging to facilitate functional characterization during infection. To accomplish this, fragment 5 was modified by in vitro CRISPR-Cas9 editing independently since it contains both of the targeted genes (fig. S5, E and F). Full-length genomes were then assembled in the p12 mutant background by TAR in yeast and confirmed by junction PCR amplification (fig. S7, E and F). Transfection of the assembled genomes resulted in red (CD2v-mCh/p12M) or blue (K145R-Cer/p12M) fluorescent–infected cell foci after infection with ΔDUT::GFP self-helper virus (fig. S8, A and B). Although the fluorescence for each mutant was sufficient for plaque purification in both cases, it was notably weaker than that observed with ASFV mutants expressing the fluorescent reporters under the control of the ASFV capsid protein p72 promoter at each targeted locus, possibly due to weaker activity of the *CD2v* and *K145R* promoters or to reduced autofluorescence or stability of the fusion proteins compared to the native reporter proteins. However, given that the viruses are viable and grow without any apparent defect, they should be useful for functional characterization of CD2v and K145R during infection.

## DISCUSSION

We have developed an innovative self-helper virus system using either a heterologous or homologous ASFV strain for live virus reconstitution from the ASFV genome. Combined with a yeast-based DNA assembly approach, our self-helper virus system establishes an ASFV reverse genetics method that enables efficient combinatorial genome-wide engineering of ASFV to facilitate the study of its biology and vaccine development. As proof of principle, we established the ASFV reverse genetics method for ASFV-Kenya-IX-1033, a genotype IX strain and demonstrated its utility by generating single, double, and triple deletion mutants targeting the *K145R*, *A238L*, and *I329L* genes. In addition, we generated strains containing fluorescent fusions of the CD2v and K145R proteins.

The self-helper virus system took advantage of our previous observation that CRISPR-Cas9 targeting of an essential gene inhibited replication of ASFV (*37*). The initial demonstration of this system with the Kenya-IX-1033 (genotype IX) virion DNA used either ArmeniaΔ285L::GFPPhuCD4 or NHVΔTK::GFP (genotype II or I, respectively) as the heterologous helper virus targeted by CRISPR-Cas9 cleavage of the *CP204L* (p30) gene locus. The p30 protein is a highly antigenic membrane–bound and secreted phosphoprotein that is transcribed early during infection (*45*, *46*). However, when we reconstituted viruses from the TAR-assembled synthetic Kenya-IX-1033 genome using the heterologous helper virus, we obtained growth-defective viruses. Thus, we also used a “homologous” helper virus strategy to boot-up the TAR-assembled synthetic Kenya-IX-1033 genome where the helper virus, Kenya-IX-1033ΔDUT::GFP was inhibited by Cas9 cleavage of the *O61R* (p12) gene locus and the TAR-assembled “synthetic genome” contained silent mutations in the *O61R* gene such that it could not be cleaved by CRISPR-Cas9. The p12 protein has been implicated in virus attachment to host cells and is transcribed late during infection (*46*, *47*). Strikingly, live virus was reconstituted successfully from transfected genomes regardless of whether an early-transcribed gene (*CP204L*) in the heterologous helper virus or a late-transcribed gene (*O61R*) in the homo-logous helper virus was targeted by CRISPR-Cas9. While it is not yet clear which genes or activities are needed for virus reconstitution from transfected genomes, it appears that both scenarios provide the relevant functions needed for live virus reconstitution. Future studies may determine the common set of transcribed genes using RNA sequencing or other methods after infection and CRISPR-Cas9 targeting of the two different genes that then can be tested as helper genes for reconstitution of live viruses from recombinant ASFV genomes.

The CRISPR-Cas system has been used previously as a means to counterselect a variety of wild-type DNA and RNA eukaryotic viruses to obtain modified recombinant viruses (*25*–*27*, *48*–*51*). It has also been used effectively for counterselection in bacteriophage and archaeal virus engineering (*52*, *53*). In these cases, the viruses are modified directly in situ after host cells are infected by introducing the desired changes that include a selection marker and/or a visualizing gene, such as a fluorescent gene, on a DNA plasmid or fragment and using the host cell’s DNA repair activity to insert them at the target locus in the infecting virus genome. The CRISPR-Cas system serves to counterselect the wild-type virus from the recombinants. Our use of the CRISPR-Cas system is distinct from these cases in that, although we are inhibiting replication of the infecting virus, there is still expression of viral proteins in the host cell and the activity of some or all of these proteins to reconstitute the synthetic recombinant virus from a different viral genome. Given these requirements, it stands to reason that any method to inhibit replication of the self-helper virus should be able to reconstitute desired recombinant viruses from modified viral genomes as long as the generated synthetic recombinant viruses are immune to the inhibition of replication and there is expression of the important viral proteins whose activities are needed to perform the virus reconstitution. Researchers in the 1960s and early 1970s demonstrated that infection of conditionally lethal “self-helper” bacteriophages greatly improved the efficiency of wild-type DNA transfection into bacterial host cells to generate live virus for a variety of bacteriophages (*38*, *54*, *55*), providing precedence for the self-helper virus idea. However, the prevailing thinking for this increase in transfection efficiency by the helper bacteriophages was primarily due to facilitating DNA entry into host cells and inhibiting intracellular inactivation of the incoming DNA by nucleases or other host defense systems, as opposed to transcribing the transfecting DNA for protein production or DNA replication for virus reconstitution (*38*, *56*).

To demonstrate the utility of our ASFV reverse genetics method, we selected the *CD2v*, *K145R*, *A238L*, and *I329L* genes for modifications and/or deletions and *K145R* as the core gene for deletion combinations for several reasons. First, each of the genes is individually nonessential, either in the genotype IX or another genotype background (*39*, *42*–*44*, *57*). In addition, the *K145R* and *A238L* genes have been deleted in combination with other genes (*42*, *58*–*60*) although not in the combinations we report in this study. Second, *K145R* is immunogenic and is a potential negative serological marker for differentiation of infected from vaccinated animals diagnostic tests (*42*, *59*, *61*). Third, *CD2v*, *A238L* and *I329L* play important roles in ASFV pathogenesis by inhibiting host antiviral pathways and innate immunity (*62*–*64*) and, hence, may be good targets to attenuate ASFV. Fourth, the genes are present in different loci distributed across the ASFV genome. Almost all of the ASFV mutant strains generated by our reverse genetics method in this study grew at comparable rates to wild-type virus in vitro. These include ΔCD2v::mCh/p12M, ΔK145R::mCh/p12M, ΔK145R::mCh/DA238L/p12M, ΔK145R::mCh/ΔI329L/p12M, and ΔK145R::mCh/ΔA238L/ΔI329L/p12M. It will be interesting to see whether these mutant strains are attenuated in vivo and whether they can provide protection against virulent challenges. Only the synthetic ΔCD2v::mCh mutant that contained partial I-SceI sites and synthetic hairpin loop sequences at its genome ends demonstrated a significant growth defect. While the reason for this defect is not clear, we speculate one below.

In addition to providing a description of a helper virus system for ASFV, our study has revealed or corroborated some important aspects of ASFV biology. One is homologous recombination, which is a driver of dsDNA virus evolution. Several studies involving sequence analysis of either specific loci or whole genomes have concluded that homologous recombination in ASFV has shaped its high degree of genetic diversity (*65*, *66*). In addition, Zhao *et al.* (*18*) recently detected three recombinants of genotype I and genotype II ASFVs in pigs in China, which were classified as genotype I due to their *B646L* gene, but contained 10 distinct fragments derived from genotype II viruses, suggesting that recombination between different genotype ASFVs occurs in nature (*18*). In support of these views, we obtained direct experimental evidence of homologous recombination in ASFV. In our series of experiments using helper virus, whether it was “heterologous or homologous,” for virus reconstitution from modified viral genomes, we observed recombination between the helper virus genome and the transfected genome at different locations. For example, in the original demonstration of the self-helper virus method using ASFV-NHVΔTK::GFP as the heterologous helper virus and the isolated Kenya-IX-1033 ΔCD2v::DsRed virion DNA as the transfected genome, we obtained a recombinant virus that contained the ASFV-NHVΔTK::GFP sequence at the p30 locus. We also obtained a couple of recombinant viruses that contained helper virus terminal sequences at least one genome end when ArmeniaΔ285L::GFPhuCD4 was used as the helper virus for virus reconstitution from a synthetic assembled ΔCD2v::mCh genome, demonstrating that recombination occurred between the two genomes at their termini. Lastly, from our combinatorial ASFV *K145R*, *A23L* and *I329L* deletion engineering efforts using the homologous helper virus, we obtained several recombinant viruses that contained wild-type *A238L* or *I329L* in combination with a *K145R* gene deletion rather than the expected deletion of either gene due to recombination between the helper virus genome and the synthetic assembled Kenya-IX-1033 genome. In addition, we obtained a number of dual fluorescent recombinant viruses due to the transfer of the ΔDUT::GFP gene from the self-helper virus to the synthetic assembled Kenya-IX-1033 genome by recombination. Although homologous recombination in ASFV is a complicating factor for our helper virus–based reverse genetics method, it is robust enough to obtain mutant viruses with the full set of expected modifications. However, our results add to the concern that recombination in ASFV poses a challenge to vaccine development based on live-attenuated viruses (*18*). The capacity of our reverse genetics method to easily generate multiple modifications across the entire ASFV genome may mitigate this challenge due to the need for multiple recombination events to restore virulence or to escape protection. Furthermore, our method could be effectively used to further study ASFV homologous recombination activity to determine hot spots and frequency to better understand its biology and diversity.

Another aspect of ASFV biology that our study elucidated more is the importance of the genomic termini, which are characterized by the presence of hairpin loops and terminal inverted repeats, for replication of the virus, similar to poxviruses, such as vaccinia virus (*41*). Gonzalez *et al.* (*41*) have determined the sequence and structure of the hairpin loops from the genotype II ASFV strain, BA71V, and highlighted sequence similarities between BA71V and vaccinia virus at termini region adjacent to the hairpin loops (*41*). The Moss laboratory has used directional deep sequencing of short single-stranded DNA fragments enriched for RNA-primed nascent strands to pinpoint the initiation of DNA replication of vaccinia virus to its hairpin loops and to provide evidence for RNA priming of lagging strand DNA synthesis at different genome locations for production of intermediate Okazaki fragments during DNA replication (*67*). ASFV appears to have a very similar mechanism for DNA replication since it also encodes a DNA primase and ligase, which likely produce intermediate Okazaki fragments and, as mentioned above, contains hairpin loops and terminal inverted repeats at the genomic termini (*68*). Thus, although it has not been investigated or determined in the ASFV genome, it is likely that ASFV DNA replication is initiated at its hairpin loops. Our results here support this view. In our experiments, we ligated synthetic hairpin loop sequences to the linear synthetic assembled genomes after they had been separated from the YCpBAC vector by RE digestion and then transfected them into WSL cells for virus reconstitution. As discussed above, when we reconstituted virus from the ΔCD2v::mCh genome using the heterologous ArmeniaΔ285L::GFPhuCD4 helper virus, we obtained growth-deficient recombinant ΔCD2v::mCh viruses that contained helper virus terminal sequences in at least one genome end through recombination, suggesting that our “artificial” genome ends in ΔCD2v::mCh were not completely functional. In line with this, from the same experiments, we did obtain one growth-deficient recombinant ΔCD2v::mCh virus that contained only Kenya-IX-1033 sequences but, with a partial I-SceI sequence between the synthetic hairpin loop and the terminal inverted repeat region. It appears that ASFV replication is quite sensitive to changes in the termini or intervening sequences between the hairpin loop and the adjacent terminal region. It is conceivable that because of limited next-generation sequencing (NGS) data of this genome region, we did not ligate the correct Kenya-IX-1033 hairpin loop sequence to the synthetic assembled genome. However, similar growth phenotypes were obtained with recombinant viruses from assembled genomes ligated with the validated BA71V hairpin loop sequences. On the other hand, we cannot discount the idea that each hairpin loop is unique to its genotype for efficient replication and that hairpin loops from other genotypes do not function properly with a genome of a different genotype. These results were in stark contrast to those obtained when we reconstituted live virus from the virion ΔCD2v::DsRed genome, which obviously contained its endogenous genome termini using the heterologous helper virus since no recombination of the termini between the two genomes was observed and whose progeny grew with wild-type kinetics. Also, as support of the view that the hairpin loops are important for ASFV replication, we obtained recombinant viruses that demonstrated wild-type growth kinetics when we used the homologous ΔDUT::GFP helper virus to reconstitute virus from assembled Kenya-IX-1033 genomes. The genomes of these resulting viruses contain the genome termini from the helper virus rather than ligated hairpin loops with partial I-SceI sequences intervening the adjacent terminal inverted repeats due to homologous recombination, restoring the natural replication sequences and leading to wild-type growth kinetics.

The synthetic genomics work presented here may raise dual use concerns since it describes methods that facilitate the engineering of an agricultural select agent. We have discussed some issues surrounding these concerns before (*32*), and they remain relevant here. However, a major dual-use concern from this work is regarding the development of a “built-in” self-helper virus system to reconstitute recombinant viruses from noninfectious genomes. The concern is that this approach can be easily adapted to other viruses, including US Federal Select Agents or emerging viruses whose genomes are not infectious to facilitate their engineering by “bad actors” for bioterrorism purposes. With respect to the US Federal Select Agent viruses, which include ASFV, approximately 50% have noninfectious genomes for which the self-helper virus system could potentially be used to reconstitute recombinant live viruses using reverse genetics. However, all of these select agent viruses can already be engineered using reverse genetics with helper genes/viruses or, like ASFV, by homologous recombination in host cells. Thus, while the combination of a self-helper virus system and a synthetic genomics assembly workflow used in this study may accelerate the generation of modified select agent viruses with noninfectious genomes, the approach does not substantially elevate this dual-use concern. Rather, we suggest that the work described here will be helpful to the greater scientific community to develop vaccines or therapeutics for pathogenic viruses or also countermeasures for those engineered for harm. Indeed, assuming that the US Federal Select Agent viruses reflect the discovery of new or emerging viruses to a first approximation, we can expect that about half will have noninfectious genomes. Hence, the methods we have established for ASFV, particularly the self-helper virus system, could be a first-in-line approach to rapidly generate engineering tools to develop countermeasures, especially for those of pandemic potential, alleviating the substantial effort needed to identify helper genes or helper viruses for live virus reconstitution. Another concern is the application of our synthetic genomics assembly techniques to recreate the genomes of other select agent viruses, such as the smallpox virus from synthetic DNA. Indeed, horsepox virus, a relative of smallpox virus, has been reconstituted from synthetic DNA (*35*). However, this capability has been available for quite some time and acknowledged by the WHO almost 10 years ago (*69*). In addition, the synthetic genomics assembly methods, applied here for ASFV, were developed for synthesis of the *Mycoplasma mycoides* genome in 2010 (*31*) and since then have been used by several groups and us for various viruses, including large DNA viruses and bacteriophages (*32*, *33*, *70*–*72*). To keep up with evolving synthetic genomics technology, the US Department of Health and Human Services (HHS) has updated its “Screening Framework Guidance,” which was first issued in 2010 (*73*) for providers and users of synthetic nucleic acids to expand the scope of its guidance to include “(i) a definition of Sequences of Concern (SOCs) that includes all sequences that contribute to pathogenicity or toxicity, whether from regulated or unregulated agents; (ii) best practices for all entities involved in the synthesis, use, and transfer of nucleic acids containing SOCs (i.e., providers and customers); (iii) best practices for manufacturers of benchtop nucleic acid synthesis equipment and institutions where they are used; (iv) recommendations for screening orders over a smaller screening window—50 nucleotides; and (v) recommendations for screening orders of all types of synthetic nucleic acids (i.e., single- and double-stranded forms of DNA and RNA)” (*74*), although these may still not be foolproof. Additional “teeth” have been added in April 2024 to the guidance through a new executive order that requires recipients of federal research funds to buy synthetic DNA only from providers who follow the HHS Guidance. Although concerns about the ability to recreate viral genomics are real, we believe that the assembly methods described here and in our previous research do not raise the level of this particular dual-use concern. We also note that a substantial amount of technical expertise and scientific equipment is still needed to accomplish the synthetic genomics workflow we have developed for various viruses, including ASFV. Therefore, it remains rather challenging for an actor with nefarious intent and even experienced virologists to abuse viral research. While we have outlined several dual-use concerns, we stress that they are offset by favorable uses of these synthetic genomics approaches to benefit public health.

In summary, we report the development of a synthetic genomics reverse genetics system for ASFV mediated by an innovative self-helper virus method. The ASFV reverse genetics system was used to generate combinatorial multi-gene deletion genotype IX strains as well as viable strains fusions of viral gene products with fluorescent proteins to facilitate development of vaccines or to better understand the biology and pathogenesis of ASFV, which is the only member of the *Asfarviridae* family (*8*). This system can easily be adapted for other ASFV genotypes, including genotype II that is now affecting many parts of the world. A TAR assembly of a clinical genotype II strain has recently been reported (*71*). In addition, it can be applied to develop reverse genetics other important agricultural pathogens, such as lumpy skin disease virus which, like ASFV, has a noninfectious dsDNA genome to accelerate vaccine or therapeutics development (*75*). The entire combinatorial genome-wide engineering approach, synthetic genomics genome assembly and built-in helper virus for viral reconstitution, can be adapted to quickly develop a reverse genetics system for emerging viruses with noninfectious genomes, especially those of pandemic concern, and even for bacteriophages, as long as the wild-type live virus is available and replication competent in cell culture.

## MATERIALS AND METHODS

### Cells, viruses, and strains

The yeast strain VL6-48N (*32*) (*MATa*, *his3-D200*, *trp1-D1*, *ura3-D1*, *lys2*, *ade2-101*, *met14*, cir**°**) was used for all yeast transformations and grown in yeast extract, peptone, and dextrose (YPD) media supplemented with adenine (4 mg/ml final concentration) and ammonium sulfate (0.5% final concentration). Yeast transformed with a YCpBAC was grown synthetic media (SD) without histidine (−HIS) or without uracil (−URA) and supplemented with adenine. *E. coli* strain DH10B (Intact Genomics) was used for production of DNA stocks of ASFV TAR clones, assembled partial genomes, and assembled complete genomes. Cultures were grown at 30°C in LB broth (Lennox) with chloramphenicol (12.5 mg/ml). ASFV-Kenya-IX-1033 and the derived mutant ASFV-Kenya-IX-1033DCD2v::dsRed, the WSL cell–adapted genotype II virus ASFV-Armenia-2008, and the derived mutant ASFV-ArmeniaΔ285L::GFPhuCD4, as well as the attenuated, cell-adapted genotype I strain ASFV-NHV, and the derived mutant ASFV-NHVDTK-GFP have been described previously (*36*, *37*, *39*, *40*, *57*, *76*). ASFV-Kenya-IX-1033ΔDUT::GFPhuCD4 was generated in a similar manner like ASFV-ArmeniaΔ285L::GFPhuCD4 by replacement of the viral dUTPase gene E165R by the same reporter gene cassette (fig. S9). The ASFV-permissive wild boar lung cell line WSL (*40*) was cultivated in Iscove’s modified Dulbecco’s medium with Ham’s F-12 nutrient mix and 10% fetal bovine serum (FBS) at 37°C in a water-saturated atmosphere containing 2.5% CO_2_. After transfection with plasmids or assembled ASFV genomes or/and infection with ASFV, the cells were maintained in medium containing only 5% FBS and 1% of a penicillin (10.000 U/ml)–streptomycin (10.000 μg/ml) solution (Thermo Fisher Scientific). For plaque assays, the infected cells were overlaid with semisolid medium additionally containing methyl cellulose (6 g/liter). The Friedrich-Loeffler-Institut is licensed by the competent German and European authorities to work with infectious wild-type and genetically engineered ASFV. All experiments with ASFV were performed in a biocontainment facility fulfilling the safety requirements for ASF laboratories and animal facilities (European Commission Decision 2003/422/EC, Chapter VIII), and biosafety level 4 standards according to the German genetic engineering safety regulations (Gentechnik-Sicherheitsverordnung, GenTSV).

### Plasmids

pCC1BAC-his3 and pCC1BAC-ura3 have been previously described (*31*). psp-ASFV-Kenia-DCD2v-FLox-p72-DsRed-ori1 has also been previously described (*37*). Fluorescent protein ORFs were amplified from pFB-ORF67mCherry (*77*) (mCherry) and pCerulean–vesicular stomatitis virus glycoprotein (VSVG) (Cerulean) (*78*). pCerulean-VSVG was a gift from J. Lippincott-Schwartz, National Institutes of Health, Bethesda, MD, USA (Addgene, plasmid # 11913), and pFB-ORF67mCherry was a gift from P. Desai, Johns Hopkins University School of Medicine, Baltimore, MD, USA.

### Yeast spheroplast preparation for transformation

Yeast spheroplasts were prepared using previously described methods (*32*, *33*, *79*). Briefly, cultures (50 ml) were grown in YPD to an optical density at 600 nm (OD_600_) of 1.8 to 2.0 and then centrifuged at 1600*g* (Eppendorf Centrifuge, 5810R) for 3 min and kept overnight at 4°C in 20 ml of 1 M sorbitol. After harvesting by centrifuging at 1600*g* for 3 min, the cells were resuspended in 10 ml of SPE solution (1 M sorbitol, 0.01 M sodium phosphate, and 0.01 M Na_2_EDTA) with 20 μl of zymolyase solution (20 mg/ml; Zymolyase 20T, US Biological Life Sciences) and 20 μl of β-mercaptoethanol (14.2 M). The cells were incubated at 37°C for 17 to 20 min with 50 rpm agitation. Good spheroplast preparation was followed during digestion by mixing 100 μl of cells with either 900 μl of 1 M sorbitol or 2% SDS and measuring OD_600_. Spheroplast preparation was considered complete when the ratio of the measurements was three to fourfold (OD_600_ 1 M sorbitol/OD_600_ 2% SDS). Once digestion was complete, 30 ml of 1 M sorbitol was added to the cells and mixed by gentle inversion. The spheroplasts were then centrifuged at 1600*g* for 5 min at 4°C. The cell pellet was resuspended gently in 20 ml of 1 M sorbitol with a 25-ml pipet, followed by addition of another 30 ml of 1 M sorbitol and mixing by gentle inversion. The spheroplasts were then centrifuged as described above. The spheroplasts were resuspended in 4 ml of storage buffer (1 M sorbitol and 15% dimethyl sulfoxide), aliquoted into 200 μl/1.5-ml tube fractions, frozen, and stored at −80°C. In our experience, the spheroplasts were competent for transformation up to a year (*79*).

### DNA transformation in yeast

Frozen spheroplasts (200 μl per tube) were thawed on ice. After thawing, 22 μl of 10× TC solution [100 mM tris-HCl (pH 7.5) and 100 mM CaCl_2_] was added to each tube and mixed by finger tapping. Each suspension was then centrifuged at 1500*g* for 5 min (Eppendorf Centrifuge, 5415R). After removal of the supernatant, each pellet was washed with 500 μl of STC solution [1 M sorbitol, 10 mM tris-HCl (pH 7.5), and 10 mM CaCl_2_] without resuspension and centrifuged as above. Each pellet was then resuspended in 200 μl of STC solution using a genomic tip. DNA for TAR cloning or assembly was added to the 200 μl of spheroplasts, incubated at room temperature for 10 min, followed by addition of 900 μl of 20% PEG solution [20% polyethylene glycol, molecular weight 8000 (PEG-8000), 10 mM CaCl_2_, and 10 mM tris-HCl (pH 7.5)]. The suspension was incubated at room temperature for 20 min, then the PEG solution was removed after centrifugation as above, and the spheroplasts were first resuspended in 200 μl of SOS media (1 M sorbitol, 6.5 mM CaCl_2_, 0.25% yeast extract, and 0.5% bactopeptone), topped to 1 ml, and then incubated at 30°C for 30 min. Transformed yeast spheroplasts were plated with 7 ml of selective top agar with sorbitol (SD media, 1 M sorbitol, and 3% bactoagar) on selective SD media with 1 M sorbitol (SD media, 1 M sorbitol, 2% and bactoagar) supplemented with adenine (4 μg/ml final concentration) and ammonium sulfate (0.5% final concentration).

### TAR cloning, screening, and processing of AFV DNA fragments

The TAR cloning, screening, and processing of the ASFV DNA fragments were performed as described in Oldfield *et al.* (*32*) with some modifications. Briefly, 800 ng of viral genomic DNA was cotransformed with 20 ng of the appropriate amplified vector (table S4) into yeast spheroplasts and selected on SD-HIS sorbitol top agar/plates supplemented with adenine (4 μg/ml final concentration) and ammonium sulfate (0.5% final concentration). Transformants were patched on SD-HIS plates supplemented with adenine (4 μg/ml final concentration) and ammonium sulfate (0.5% final concentration) and grown for at least 2 days at 30°C. Yeast from the patches was picked into 10 μl of 25 mM NaOH and incubated at 95°C for 30 min to lyse yeast cells. PCR amplification with Q5 high-fidelity DNA polymerase, according to the manufacturer’s instructions (New England Biolabs, catalog no. M0491L), on DNA isolated from lysed yeast was used to confirm the correct junction of the vector and ASFV fragment on either side using the appropriate set of primers (table S5, RCO495 and fragment detection 5p primer, RCO493 and fragment detection 3p primer).

Positive TAR clone candidates from yeast patches were resuspended in 500 μl of water containing 5 μl of zymolyase 20T (MP Bio, 10 mg/ml) and 0.5 μl of β-mercaptoethanol (14.2 M) and incubated at 37°C for 1 hour. Fifty microliters of 2% SDS was added, and the solution was incubated for 15 min at 70°C, followed by addition of 50 μl of 5 M potassium acetate and incubation on ice for 5 min. An equal volume of phenol:chloroform:isoamyl alcohol (25:24:1) was added, mixed, and centrifuged at 16,100*g* for 10 min (Eppendorf Centrifuge, 5415R). After transfer of the clear top aqueous phase to a new microfuge tube, the DNA was precipitated with an equal volume of isopropanol followed by centrifugation at 16,100*g* for 15 min (Eppendorf Centrifuge, 5415R) and resuspended in 50 μl of TE with incubation at 37°C for 15 min. The isolated DNA was then electroporated into DH10B *E. coli*–competent cells and selected on LB plates with chloramphenicol (12.5 μg/ml) at 30°C overnight. Transformants were confirmed for the appropriate vector/fragment junctions by growing the colonies in 1 ml of Terrific Broth (Invitrogen) with chloramphenicol (12.5 μg/ml) at 30°C overnight, pelleting 100 μl of the growth (VWR Galaxy mini, 5 min at room temperature), resuspending the pellet in 10 μl of 25 mM NaOH with incubation at 95°C for 30 min to lyse the cells and PCR amplification as above.

### TAR assembly of partial (third and half) and complete genomes from ASFV DNA fragments

ASFV partial and complete genomes were performed as described in Vashee *et al.* (*33*) with some modifications. Briefly, cultures for all 12 ASFV fragment clones in *E. coli* were grown up and DNA was isolated using the PureLink HiPure Plasmid Midiprep kit (Thermo Fisher Scientific). Before assembly, the clones were digested with I-SceI and heat-killed to release the ASFV fragments from the YCpBAC. TAR assembly of ASFV genomes was performed in two steps. First, one-third genomes (fragments 1 to 4, fragments 5 to 8, and fragments 9 to 12) were assembled as follows. For one-third genomes, vector DNA was amplified using pCC1BAC-ura3 as the template and either Con_01_5p and Con_04_3p as primers for fragments 1 to 4 or Con_05_5p, Con_08_3p for fragments 5 to 8, or Con_09_5p and Con_12_3p for fragments 9 to 12. For assembly of each partial genome, 20 ng of appropriate amplified vector and 800 ng of each of the pertinent I-SceI–digested fragments was transformed into yeast spheroplasts with selection on SD-URA sorbitol top agar/plates supplemented with adenine (4 μg/ml final concentration) and ammonium sulfate (0.5% final concentration). Transformants were patched onto SD-URA plates supplemented with adenine (4 μg/ml final concentration) and ammonium sulfate (0.5% final concentration), and DNA was screened by PCR amplification for the appropriate junctions with the respective detection primers (table S5) using NaOH lysis as described above. Positive third and half genomes were transformed into *E. coli* DH10B–competent cells and screened by PCR amplification to confirm the appropriate junctions. For assembly of the complete genome, the one-third genomes were processed with I-SceI as described above to release the fragments. Vector DNA was amplified by PCR using pCCIBAC-his3 as the template and Con_01_5p and Con_12_3p as primers. Twenty nanograms of amplified vector and 800 ng of each third or half genome were transformed into spheroplasts with selection on SD-HIS sorbitol top agar/plates supplemented with adenine (4 μg/ml final concentration) and ammonium sulfate (0.5% final concentration). Transformants were patched onto SD-HIS plates supplemented with adenine (4 μg/ml final concentration), and DNA was screened by PCR amplification for the appropriate junctions with the respective detection primers (table S5) using NaOH lysis as described above. As before, positive clones were transformed into *E. coli* DH10B–competent cells and screened by PCR amplification to confirm the appropriate junctions.

### In vitro CRISPR-Cas9 editing of ASFV TAR clones

Modification of ASFV TAR clones were performed using in vitro CRISPR-Cas9 editing as described in Oldfield *et al.* (*32*) with some modifications. Briefly, targets for the sgRNA contain 12 bp of unique sequence upstream of a PAM sequence. A linear DNA template for the sgRNA was generated by PCR amplification from an ultramer template (CRISPR ultramer) containing the sgRNA scaffold sequence, which was amplified with a forward primer with a T7 promoter and the 20-bp target sequence upstream of the PAM and a reverse primer (CRISPR R) (table S5). The sgRNA was produced by in vitro transcription with the HI Scribe T7 kit (New England Biolabs). Transcription was performed with approximately 200 ng of DNA template, per the manufacturer’s instructions, except that transcription was carried out at 37°C for 2 hours. RNA was purified using the RNA Clean & Concentrator-5 kit (Zymo Research, catalog no. R1016) according to the manufacturer’s instructions and quantified by gel electrophoresis. Digestion of the desired ASFV TAR clone DNA was carried out in vitro with Cas9 nuclease, *Streptococcus pyogenes* (New England Biolabs), per the manufacturer’s instructions.

A DNA “fix” was used to repair the CRISPR-Cas9–digested ASFV TAR clone DNA. The fix contained 40 to 60 bp of homology sequence to the TAR clone beyond the Cas9 cleavage site and desired mutations or insertions at the site of cleavage. In the case that the CRISPR-Cas9 cleavage site was some distance from the desired site of modification (due to PAM site location), nucleotides were included in the fix to repair the off-center cleavage. CRISPR-Cas9–digested TAR clones and the DNA fix were assembled using TAR assembly in yeast as described above.

### Replacements, deletions and fluorescent gene fusions of ASFV genes in ASFV TAR clones

The ASFV *CD2v* gene was replaced with dsRed in fragment 5 as follows. Cas9 was used to cut fragment 5 DNA in vitro at coordinates 70,528 and 71,379 as described above. A DNA fix was generated by PCR amplification from psp-ASFV-Kenia-DCD2v-FLox-p72-DsRed-ori1 (*37*) using primers ASFV CD2v-dsRed 5′ and ASFV CD2v-dsRed 3′ (table S8). The CRISPR-Cas9–cleaved fragment 5 and DNA fix were assembled by TAR in yeast as described above and results in a replacement of *CD2v* with dsRed between coordinates 70,557 and 71,443. Positive clones were identified by colony PCR amplification using primers ASFV CD2v Del 5′ Det and ASFV CD2v Del 3′ Det (table S8) and then transferred to *E. coli* for DNA purification. The ASFV CD2v gene was replaced with mCherry in fragment 5 as follows. A DNA fix was generated by PCR amplification from pFB-ORF67mCherry (*77*) using primers ASFV Flu Rep 3′ Fix 5′ and ASFV CD2v Flu Rep 3′ Fix 3′ (table S8). The CRISPR-Cas9–cleaved fragment 5, DNA fix and oligo, ASFV CD2v Flu Rep 5′ Fix (table S8), were assembled by TAR in yeast as described above and results in a replacement of *CD2v* with mCherry between coordinates 70,324 and 71,443. Positive clones were identified and transferred to *E. coli* as described above. The ASFV CD2v-mCherry fusion was generated as follows. Two overlapping DNA fixes were generated by PCR amplification. One (to amplify a CD2v fragment) was generated from fragment 05 DNA using primers ASFV CD2v mCh Fusion Fix 5′ and ASFV CD2v mCh Fusion Fix 3′, whereas the other (to amplify mCherry) was generated from pFB-ORF67mCherry (*77*) using primers ASFV Cd2v mCh Fusion 5′ and ASFV Cd2v mCh Fusion 3′ (table S8). The CRISPR-Cas9–cleaved fragment 5 and DNA fixes were assembled by TAR in yeast as described above. Positive clones were identified and transferred to *E. coli* as described above. The modifications were confirmed by Sanger sequencing.

The ASFV *K145R* gene was replaced with mCherry in fragment 5 as follows. Cas9 was used to cut fragment 5 DNA in vitro at coordinates 61,765 and 61,904 as described above. A DNA fix was generated by PCR amplification from pFB-ORF67mCherry (*77*) using primers ASFV Flu Rep 3′ Fix 5′ and ASFV K145R Flu Rep 3′ Fix 3′ (table S8). The CRISPR-Cas9–cleaved fragment 5, DNA fix and oligo, ASFV K145R Flu Rep 5′ Fix (table S8), were assembled by TAR in yeast as described above and results in a replacement of *K145R* with mCherry between coordinates 61,722 and 62,161. Positive clones were identified by colony PCR amplification using primers, RCO495 (table S5) and ASFV K145R Del 3′ Det (table S8) and transferred to *E. coli* for DNA purification. The ASFV K145R-Cerulean fusion was generated as follows. Two overlapping DNA fixes were generated by PCR amplification. One (to amplify a K145R fragment) was generated from fragment 5 DNA using primers ASFV K145R Cerulean Fusion Fix 5′ and ASFV K145R Cerulean Fusion Fix 3′, whereas the other (to amplify Cerulean) was generated from pCerulean-VSVG (*78*) using primers ASFV K145R Cerulean Fusion 5′ and ASFV K145R Cerulean Fusion 3′ (table S8). The CRISPR-Cas9–cleaved fragment 05 and DNA fixes were assembled by TAR in yeast as described above. Positive clones were identified and transferred to *E. coli* as described above. The modifications were confirmed by Sanger sequencing.

The ASFV *A238L* gene was deleted in fragment 4 as follows. Cas9 was used to cut fragment 4 DNA in vitro at coordinate 48,093 as described above. The CRISPR-Cas9–cleaved fragment 4 and oligo, ASFV A238L Del Fix (table S8), were assembled by TAR in yeast as described above and results in a deletion of *A238L* between coordinates 47,420 and 48,141. Positive clones were identified by colony PCR amplification using primers ASFV A238L Del 5′ Det and ASFV A238L Del 3′ Det (table S8) and transferred to *E. coli* for DNA purification. The deletion was confirmed by Sanger sequencing.

The ASFV *I329L* gene was deleted in fragment 12 as follows. Cas9 was used to cut fragment 12 DNA in vitro at coordinates 170,454 and 171,000 and as described above. The CRISPR-Cas9–cleaved fragment 12 and oligo, ASFV I329L Del Fix (table S8), were assembled by TAR in yeast as described above and results in a deletion of *I329L* between coordinates 170,185 and 171,176. Positive clones were identified by colony PCR amplification using primers ASFV I329L Del 5′ Det and ASFV I329L Del 3′ Det (table S8) and transferred to *E. coli* for DNA purification. The deletion was confirmed by Sanger sequencing.

The *O61R* gene in fragment 9 was modified as follows. Cas9 was used to cut fragment 9 DNA in vitro at coordinate 126,348 as described above. The CRISPR-Cas9–cleaved fragment 9 and oligo, ASFV O61R Change Fix (table S8), were assembled by TAR in yeast as described above and results in a modification of *O61R* between coordinates 126,330 and 126,347. Positive clones were identified by colony PCR amplification using primers ASFV O61R Change 5′ Det and ASFV O61R Change 3′ Det (table S8) and transferred to *E. coli* for DNA purification. The modification was confirmed by Sanger sequencing.

### Reconstitution of ASFV from genomic virion DNA and from synthetic genomes

YCpBAC DNA was digested with I*-Sce*I to release the assembled and differentially mutated ASFV complete genomes, and then optionally, synthetic oligonucleotides forming terminal hairpin loops were ligated to the sticky fragment ends (table S6). WSL cells were grown overnight to subconfluent monolayers in 12-well plates and transfected with approximately 1 μg per well of genomic ASFV DNA, or digested YCpBAC DNA using K2 Transfection System (Biontex) as recommended by the manufacturer but including a 1-hour centrifugation of the plate at 25°C and 690*g* after DNA addition. After 6 hours, the transfection solution was removed, and cells were infected with heterologous (e.g., ASFV-Armenia-2008 derivates) or homologous (ASFV-Kenya-IX-1033 derivates) helper viruses at an MOI of approx. 1 by centrifugation for 1 hour at 37°C and 690*g*. Then, the inoculum was replaced by fresh medium, and the cells were incubated at 37°C until cytopathic effect (CPE) and optionally green, red, or blue fluorescence became visible (approx. 5 days). After freeze-thawing of the cultures, serial dilutions of the virus progenies were analyzed by plaque assays on WSL cells. Single foci of fluorescent-infected cells were identified by microscopy, labeled, and aspirated, and plaque purification was repeated until the virus populations appeared homogeneous (approximately three times).

### Preparation of ASFV DNA

WSL cells were grown in tissue culture flasks (25 to 175 cm^2^) to subconfluent monolayers and infected at low MOI with the analyzed ASFV mutants. After development of pronounced CPE (approximately 7 days postinfection), the cell cultures were lysed by freeze-thawing, and centrifuged for 10 min at 2000*g* and 4°C. The supernatants were stored as virus stocks at −80°C, whereas the cell pellets were used for preparation of total DNA as described (*80*).

For preparation of high purity ASFV DNA for transfection experiments and NGS, 100 ml of virus supernatants were centrifuged in a Beckman SW32 rotor for 1 hour at 20,000 rpm and 4°C. The pellets were resuspended in in a total amount of 1 ml TE (pH 7.4), and, after addition of 10 μl of 1 M MgCl_2_, 20 μl of deoxyribonuclease I (5 U/μl), and 10 μl of ribonuclease A (10 μg/μl), incubated for 1 hour at 37°C. In an SW32 tube, 30 ml of phosphate-buffered saline (PBS) were under-layered with 6 ml of 30% sucrose in PBS, and the nuclease-treated particles were added on top. After centrifugation for 2 hours at 20,000 rpm and 4°C, the supernatant was completely aspirated, and the pellet was used for DNA preparation as above (*80*).

### PCR and sequence analyses of ASFV DNA

Genome regions of interest of the obtained ASFV mutants were amplified by PCR using KOD Xtreme Hot Start DNA Polymerase (Merck) and specific primer pairs (tables S5 and S9). The regions amplified were the terminal inverted repeat sequences (nucleotide range in ASFV-Kenya-IX-1033: nt. 135 to 774 and 186,242 to 186,881; using primers AKT-PSF/AKT-PSR, table S9), the *K196R* (TK) gene region (nt. 61,143 to 61,878; using primers Det_04_3p/Det_05_5p, table S5), the *B962L* gene region (nt. 89,925 to 90,544; using primers Det_06_3p/Det_07_5p, table S5), the *CP240L* (p30) gene region (nt. 122,143 to 122,823; using primers CP204L-PSF/CP2404L-PSR, table S9), and the E199L (j18L) gene region (nt. 163,253 to 164,057, using primers ASFVKE199L-F/ASFVKE199L-R, table S9). After separation on agarose gels, the PCR products were isolated (Zymoclean Gel DNA Recovery Kit, Zymo Research) and investigated by Sanger sequencing using the BigDye Terminator v1.1 Cycle Sequencing Kit (Thermo Fisher Scientific) and the respective amplification primers. DNA sequences were determined in an Applied Biosystems 3500 Genetic Analyzer (Thermo Fisher Scientific) and evaluated using the Geneious Prime 2021.0.1 software package (Biomatters, available from https://geneious.com).

The complete genome sequences of the booted-up ASFV-Kenya-IX-1033ΔCD2v::DsRed (with heterologous self-helper virus), ASFV-Kenya-IX-1033ΔCD2v::mCherry (with heterologous and homologous self-helper virus), and ASFV-Kenya-IX-1033ΔK145R::mCherry/ΔA238L/ ΔI329L/p12 (homologous self-helper virus) clones as well as the conventionally generated mutant ASFV-Kenya-IX-1033ΔDUT::GFPhuCD4 were determined from purified virion DNA by custom Illumina NovaSeq NGS (Eurofins Genomics), yielding approximately 675,000 to 2.6 million pairs of ASFV-specific 150 nt reads, resulting in a mean coverage of >1200 reads per genome position (tables S2 and S7). The reads were assembled using the Geneious Prime program “Map to Reference” and the in silico mutagenized genome sequence of ASFV-Kenya 1033 (*36*). The assembled and annotated sequences will be deposited in GenBank.

### Autofluorescence analyses and plaque size determination

WSL cell monolayers grown on 24-well plates were infected with serial dilutions of wild-type or mutant ASFV and centrifuged for 1 hour at 690*g* at 37°C. For plaque assays, the inoculum was replaced by semisolid methyl cellulose medium, and the cells were incubated for 4 days at 37°C. Autofluorescence of GFP, mCherrry, or Cerulean was analyzed using a Leica DMi8 motorized fluorescence microscope and Leica Application Suite X software. For plaque size determination, whole wells were imaged using the automated well coverage function, and the resulting mosaic images were merged. For each virus, the areas of ≥50 plaques from two independent experiments were determined using ImageJ (https://imagej.net/ij/). Mean relative sizes were compared to plaques of wild-type–like spreading ASFV-Kenya-IX-1033ΔCD2v::DsRed or ASFV-Kenya-IX-1033ΔDUT::GFPhuCD4, which were set at 100%, and standard deviations were calculated. The statistical significance of differences was estimated by two-sided Student’s *t* tests.

### Virus replication kinetics

Virus replication kinetics were performed on WSL cells grown overnight to confluent monolayers in 24-well plates. The cells were infected at an MOI of approx. 0.03 with all investigated ASFV variants in three replicates for each incubation time. Infection was synchronized by centrifugation of the plates for 1 hour at 690*g* and 37°C. Then, the inoculum was replaced by fresh medium containing penicillin and streptomycin, and single plates were frozen at −80°C immediately thereafter, and after 24, 48, 72, 120, and 168 hours at 37°C. After thawing, the lysates were serially diluted, and virus was titrated by plaque assays on WSL cells grown in 96-well plates as described above. After 4 to 5 days at 37°C, the cells and the autofluorescent plaques in appropriate wells were counted. Mean virus titers of the three replicates of each mutant at each time were calculated and plotted.
