# Supplementary material for: A synthetic genomics-based African swine fever virus engineering platform
Source: Sci Adv. 2025 Mar 26;11(13):eadu7670. doi: 10.1126/sciadv.adu7670 (PMC11939070; doi:10.1126/sciadv.adu7670)
Supplement: Supplementary file 1 — Supplementary Text Figs. S1 to S9 Tables S1 to S9 Legends for data S1 to S5 [file sciadv.adu7670_sm.pdf]

Science Advances
AAAS

Supplementary Materials for

A synthetic genomics-based African swine fever virus engineering platform

Walter Fuchs *et al.*

Corresponding author: Sanjay Vashee, svashee@jcvi.org

Sci. Adv. **11**, eadu7670 (2025)
DOI: 10.1126/sciadv.adu7670

The PDF file includes:

Supplementary Text
Figs. S1 to S9
Tables S1 to S9
Legends for data S1 to S5

Other Supplementary Material for this manuscript includes the following:

Data S1 to S5

We established a synthetic genomics genome assembly process to assemble ASFV genomes, analogous to one that we previously reported for human herpesviruses *(32, 33)*. This process involved the *in silico* deconstruction of the Kenya-IX-1033 genome into 12 overlapping fragments or "parts" ranging from 12-22 kilobases in size (**Figure 2A, Table S3**), based on the recently determined sequence (GenBank # OZ005801). Except for the repeat genome ends, all of the overlapping regions between the fragments were unique to enable cloning of the individual parts and subsequent assembly by TAR in yeast. Each overlapping part was cloned by first amplifying a yeast centromeric plasmid containing bacterial artificial chromosome (BAC) sequence (YCpBAC) using pairs of primers containing 45 base pair (bp) "TAR hooks" that are homologous to the ends of each target fragment (**Table S4**) and then transforming it into yeast spheroplasts together with Kenya-IX-1033 viral DNA (**Figure S2A**). Each part contains 80 bp of homology to its adjacent parts as well as an I-SceI site on either side. Correct candidates of each part were identified by junction PCR amplification (**Table S5**), transformed into *E. coli* and then characterized by restriction enzyme analysis, as shown for DNA Fragment 5 as an example (**Figure S2B and S2C**). In addition, each wild-type part used in subsequent assembly steps was confirmed by sequencing. The efficiency of cloning the Kenya-IX-1033 parts ranged from 6% (Fragment 5) to 50% (Fragment 11).

The full-length ASFV-Kenya 1033 "synthetic" genome was assembled from the wild-type parts in two steps. First, the three individual one-third-genomes (1-4, 5-8 and 9-12) were assembled by digesting the individual TAR parts with I-SceI to release the ASFV fragments from the YCpBAC (**Figure S3A**) and then co-transforming overlapping DNA Fragments 1 to 4, Fragments 5 to 8 and Fragments 9-12, respectively, into yeast together with the YCpBAC vector containing the

appropriate TAR hooks (**Figure 2B, Table S4**). As in the previous step, the resulting correctly assembled third-genomes, identified by junction PCR amplification, were transformed into *E. coli* and characterized by junction PCR analysis (**Figure S3B**). The assembly efficiencies were higher than 50% of the clones tested. Second, full-length genomes were assembled by releasing the third-genomes from the YCpBAC backbones by I-SceI restriction enzyme digestion (**Figure S3C**) and transforming the relevant parts into yeast together with a linearized YCpBAC containing TAR hooks for the beginning and end of the Kenya-IX-1033 genome. The resulting full-length genomes in yeast were identified by junction PCR amplification and positive clones were transformed into *E. coli* where they were also characterized for full-length by junction PCR amplification (**Figure S3D**). We have obtained full-length genome assembly efficiencies in yeast as high as 100% of clones tested.

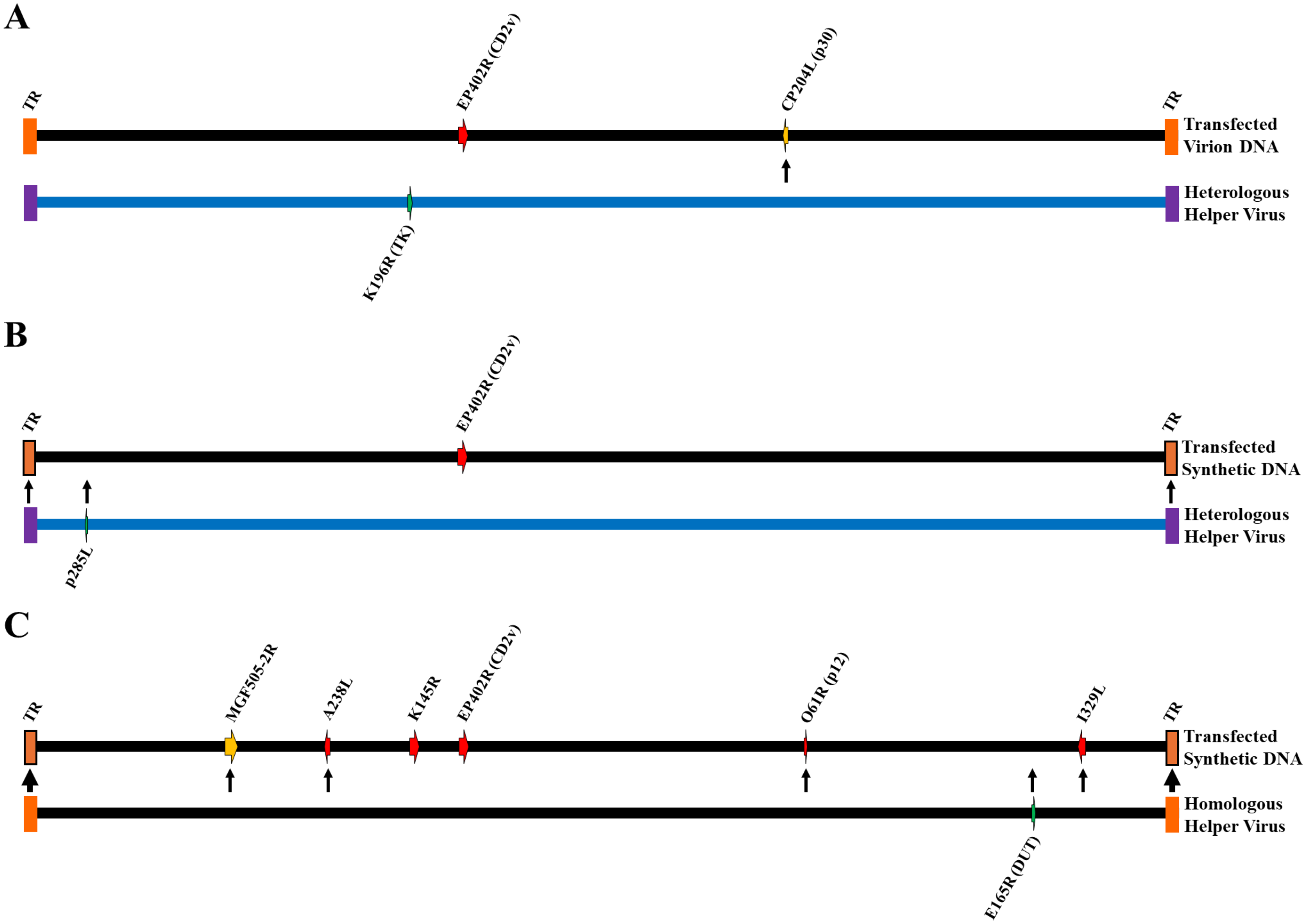

Fig. S1. Summary of recombination loci of self-helper virus-reconstituted ASFV mutants. The frequency of recombination observed during virus reconstitution of recombinant ASFV varied, depending on the helper virus and the DNA used for transfection. **(A)** Recombination was barely observed during virus reconstitution with a "heterologous" helper virus and transfected virion DNA. Helper virus sequences were only noted in gene CP204L in a single recombinant virus clone out of 10 analyzed. **(B)** However, recombination frequencies increased during virus reconstitution with transfected "synthetic" assembled ASFV genomes and a "heterologous" helper virus with the observation of numerous green, double-fluorescent and red plaques. Out of 3 red virus clones sequenced, 2 clones contained helper virus sequences at least as one of the repetitive genome ends (TR). **(C)** As expected, highest recombination frequencies were observed with the "homologous" helper virus and transfected "synthetic" assembled ASFV genomes. It is important to note that our study, which sequenced certain variable genome locations in some pertinent plaque progeny and the genomes of a few relevant ASFV recombinant mutants, does not precisely identify the sites of the recombination events but only genes or genome ends where modifications were noted **(represented by black arrows).** An additional important point is that recombination at the TRs were beneficial in the virus reconstitution from "synthetic" assembled genomes with homologous helper virus since they restored fully functional wild-type genome ends instead of the likely compromised artificial genome ends that were ligated to the assembled genomes in all of the virus isolates we analyzed (**represented by large black arrows**). The genes targeted for deletion, substitution or modification are represented by **red arrows.** The **green arrows** represent the modified genes in either the "heterologous" or "homologous" helper virus and the **gold arrows** represent genes where unexpected recombination events were observed in various virus reconstitution experiments. The TRs for the different ASFV genomes have different colors to represent their differences in sequence. The TRs for transfecting Kenya virion genome and the homologous helper virus are represented by **orange bars** whereas the TRs for the "heterologous" helper virus are represented by **purple bars** and for the synthetic genomes, they are represented by **black rectangle bordered-orange bars** to reflect the artificial hairpin loops ligated to the ends of the assembled genomes.

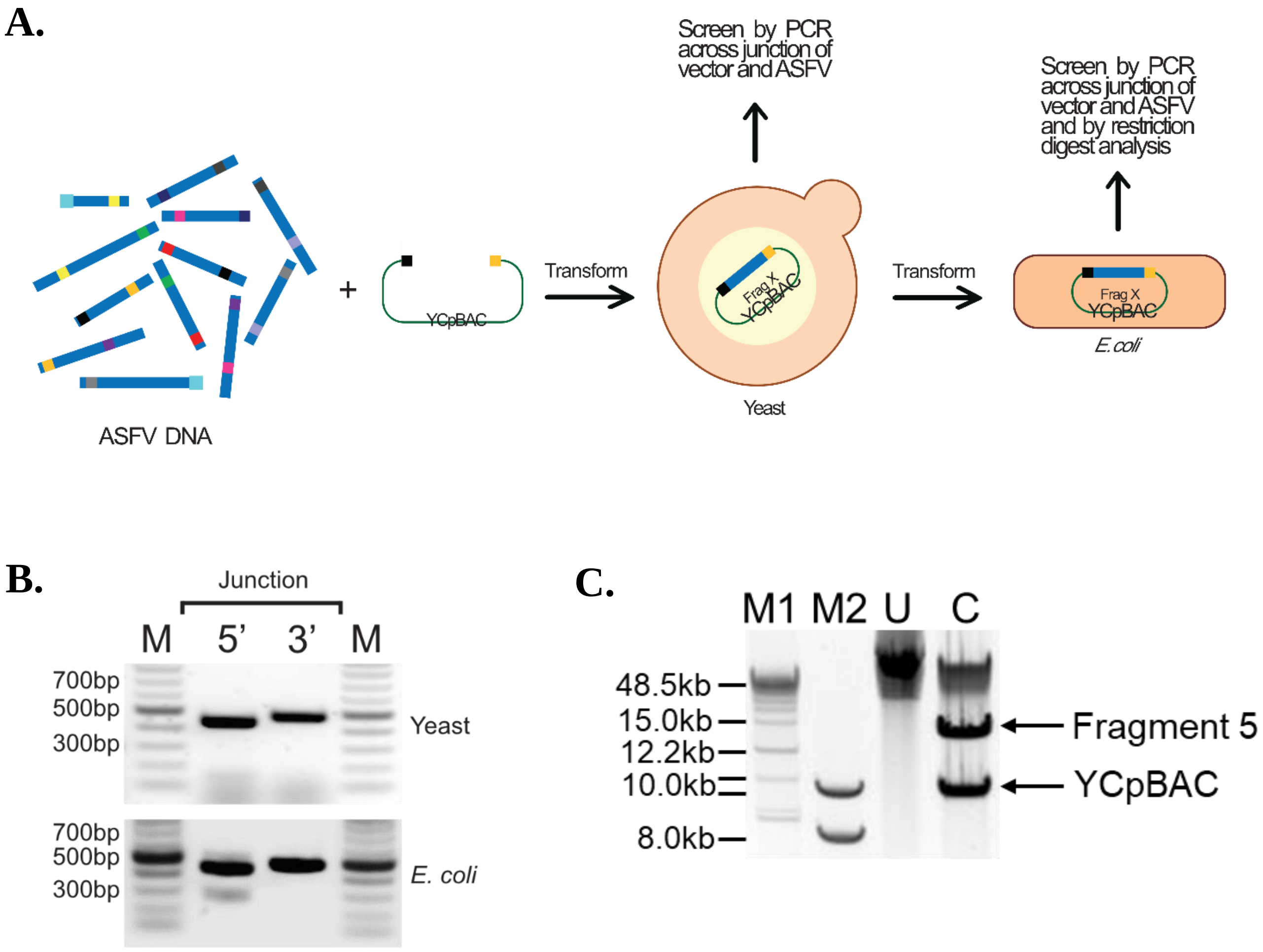

Fig. S2. TAR cloning of ASFV-Kenya-IX-1033 fragments. A. Diagram of TAR cloning of ASFV fragments from ASFV-Kenya-IX-1033 virus DNA. ASFV genomic DNA was co-transformed with a linear PCR-amplified YCpBAC vector containing "hooks" (colored boxes) with 45 bp of homology to the desired ASFV fragment and flanking I-SceI restriction sites into yeast cells. Yeast transformants were screened by PCR amplification and positive clones were then transferred to *E. coli*. *E. coli* transformants were also screened by PCR amplification and restriction enzyme digestion. **B.** A representative agarose gel after PCR amplification showing the presence of the 5' and 3' junctions of the cloned ASFV Fragment 5 in yeast and *E. coli*. **C.** A representative agarose gel after I-SceI digestion of the cloned ASFV Fragment 5 DNA. The ASFV Fragment 5 was released from the YCpBAC vector after digestion and can be used for subsequent one-third genome assembly reactions. M1, 8-48kb DNA ladder (Bio-Rad). M2, 1kb Plus DNA ladder (New England Biolabs). U, uncut DNA. C, DNA cleaved by I-SceI.

A.

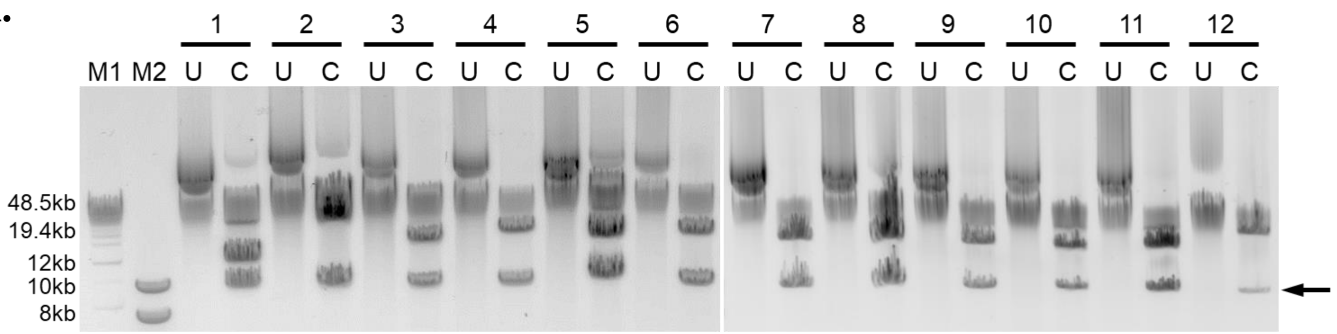
1
2
3
4
5
6
7
8
9
10
11
12
M1 M2 U C U C U C U C U C U C U C U C U C U C U C U C
48.5kb
19.4kb
12kb
10kb
8kb

B.

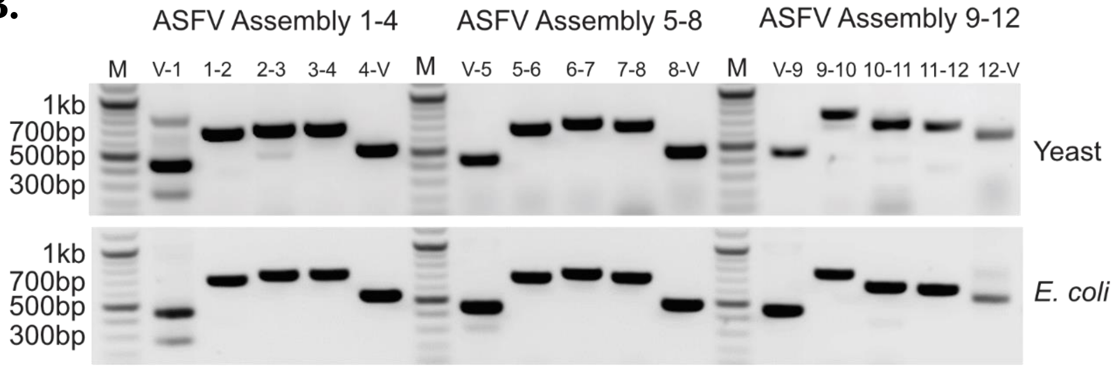
ASFV Assembly 1-4
ASFV Assembly 5-8
ASFV Assembly 9-12
M V-1 1-2 2-3 3-4 4-V M V-5 5-6 6-7 7-8 8-V M V-9 9-10 10-11 11-12 12-V
1kb
700bp
500bp
300bp
Yeast
1kb
700bp
500bp
300bp
E. coli

C.

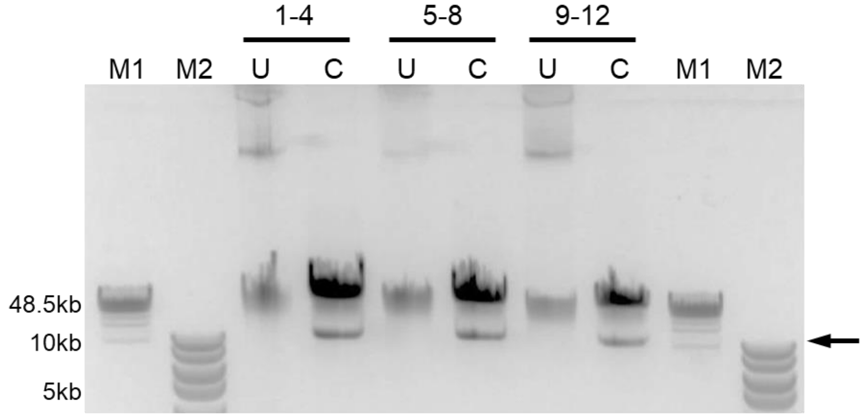
1-4
5-8
9-12
M1 M2 U C U C U C M1 M2
48.5kb
10kb
5kb

D.

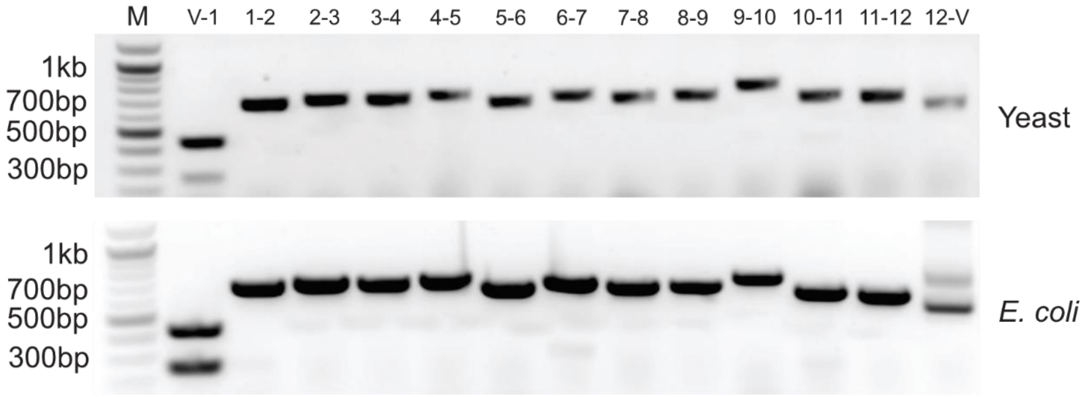

Fig. S3. Assembly of wild-type ASFV one-third and full-length genomes. Assembly of a wild-type ASFV full-length genome was performed by first assembling one-third genomes from I-SceI-cleaved wild-type parts by TAR in yeast followed by transfer of positive clones into *E. coli*. Then, full-length genomes were assembled from the I-SceI-cleaved one-third genomes by TAR in yeast followed again by transfer to *E. coli*. The assemblies were screened at each step by junction PCR amplification in both yeast and *E. coli*. **A.** A representative agarose gel after I-SceI digestion of the TAR-cloned ASFV parts (1-12) showing release of the individual fragments from the YCpBAC vector (indicated by arrow). **B.** A representative agarose gel after PCR amplification showing the presence of all appropriate junctions in the different on-third genomes. **C.** A representative agarose gel after I-SceI digestion of the TAR-assembled one-third ASFV genomes (1-4, 5-8 and 9-12) showing release of the individual one-third genomes from the YCpBAC vector (indicated by arrow). **D.** A representative agarose gel after PCR amplification showing the presence of all appropriate junctions in the full-length genome. For **A** and **C,** M1, 8-48kb marker (Bio-Rad). M2, 10kb ladder (New England Biolabs). U, uncut DNA. C, DNA cleaved by I-SceI while for **B** and **D,** M. 10kb ladder (New England Biolabs).

A.

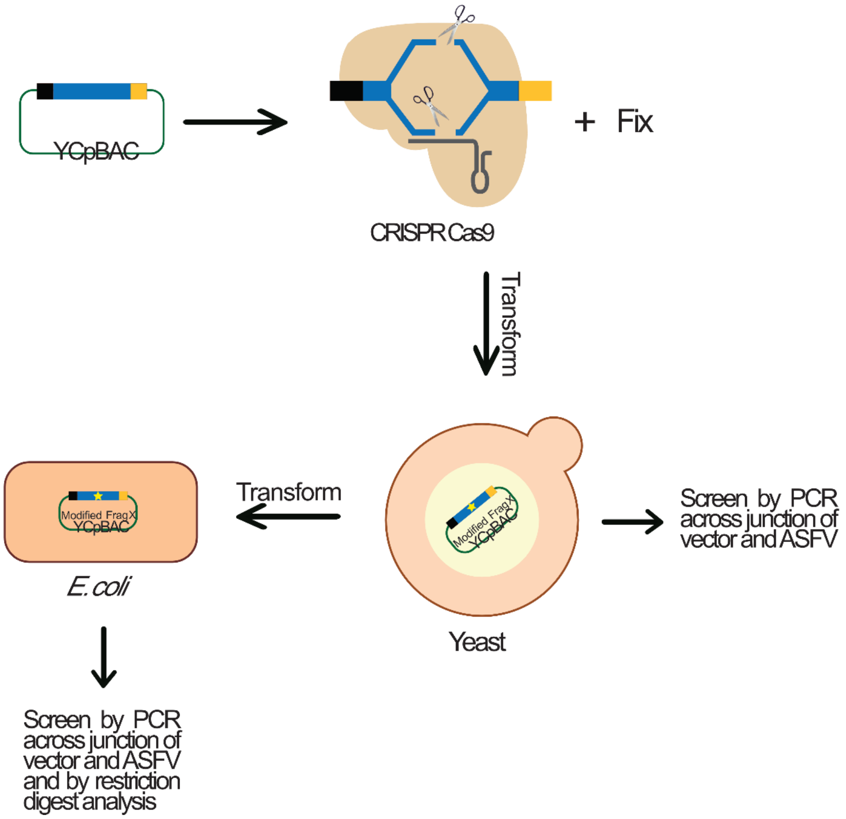
YCpBAC
CRISPR Cas9
+ Fix
Transform
Modified FragX
YCpBAC
Yeast
Screen by PCR across junction of vector and ASFV
Transform
Modified FragX
YCpBAC
E. coli
Screen by PCR across junction of vector and ASFV and by restriction digest analysis

B.

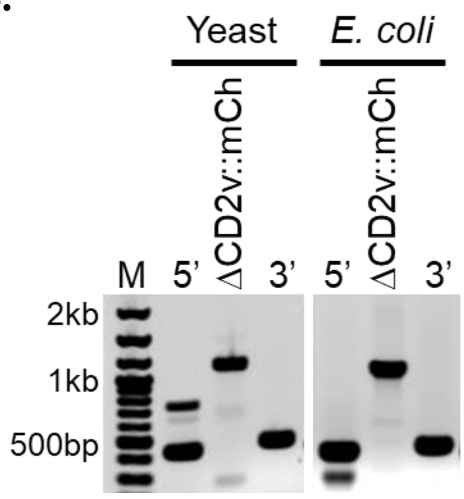
Yeast
E. coli
M
5'
ΔCD2v::mCh
3'
5'
ΔCD2v::mCh
3'
2kb
1kb
500bp

Fig. S4. Replacement of the ASFV CD2v gene with mCherry fluorescent gene. A. Diagram of CRISPR-Cas9-based modification of ASFV Fragment 5 to replace the CD2v gene with the fluorescent mCherry gene expressed under control of the ASFV p72 promoter. Fragment 5 DNA was cleaved *in vitro* by Cas9 nuclease guided by 2 CD2v-specific gRNAs and then co-transformed into yeast cells together with a "fix" (containing the fluorescent mCherry gene driven by the p72 promoter) flanked by 45 bp of homology to either side of the CD2v locus. Yeast transformants were screened by PCR amplification and positive clones were then transferred to *E. coli*. *E. coli* transformants were also screened by PCR amplification and restriction enzyme digestion. **B.** A representative agarose gel after PCR amplification showing the presence of the mCherry gene as well as the 5' and 3' junctions of the cloned ASFV Fragment 5 in yeast and *E. coli*. The 5' PCR amplification product in yeast corresponds to the lower band.

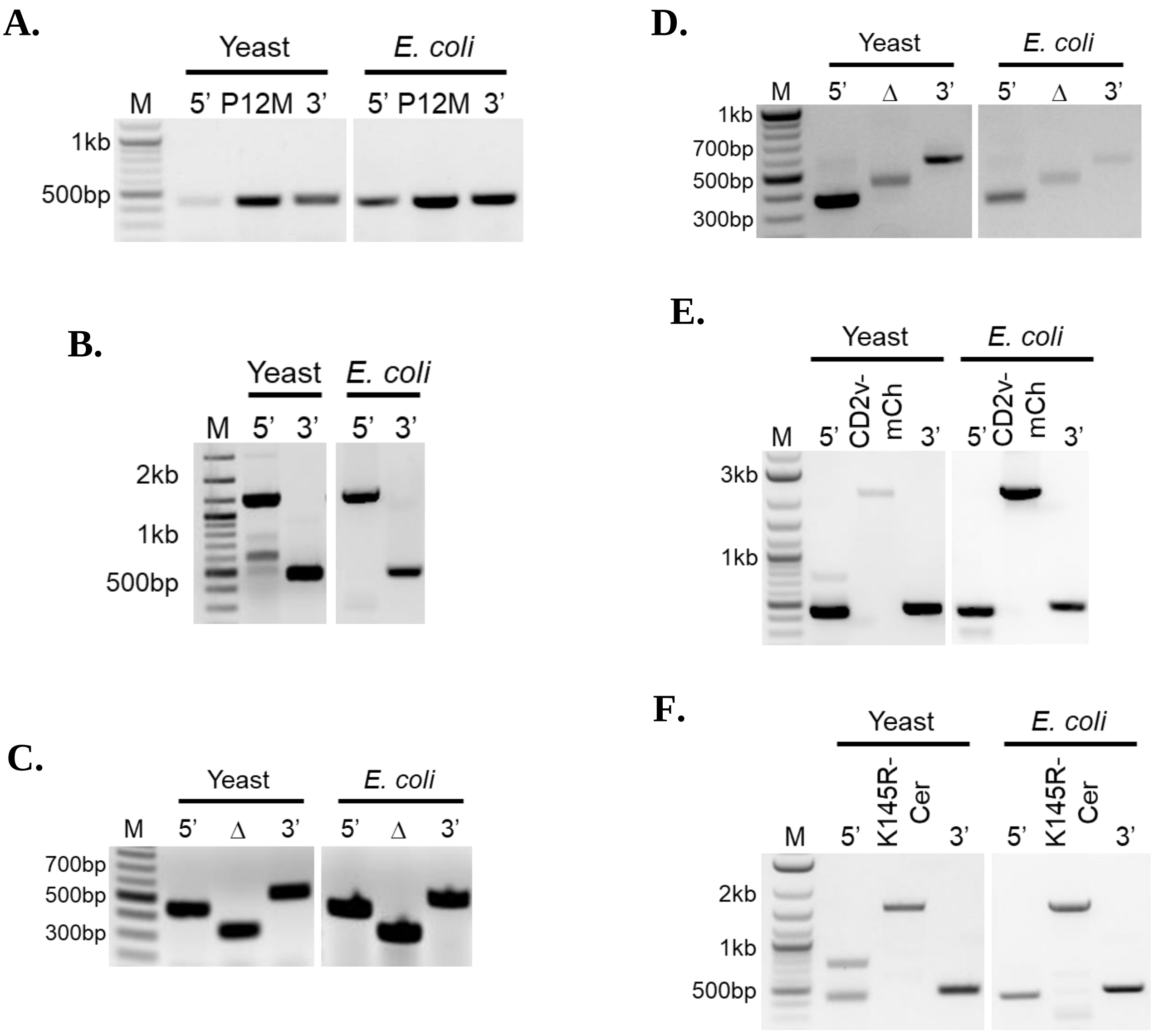
A.
Yeast
E. coli
M
5’ P12M 3’
5’ P12M 3’
1kb
500bp
B.
Yeast
E. coli
M 5’ 3’ 5’ 3’
2kb
1kb
500bp
C.
Yeast
E. coli
M 5’ Δ 3’ 5’ Δ 3’
700bp
500bp
300bp
D.
Yeast
E. coli
M 5’ Δ 3’ 5’ Δ 3’
1kb
700bp
500bp
300bp
E.
Yeast
E. coli
M 5’ CD2v-mCh 3’ 5’ CD2v-mCh 3’
3kb
1kb
F.
Yeast
E. coli
M 5’ K145R-Cer 3’ 5’ K145R-Cer 3’
2kb
1kb
500bp

Fig. S5. Deletion, replacement, modification or fluorescent gene-fusions of ASFV genes. The ASFV genes, O61R (P12), K145R, A238L and I329L genes were targeted for modifications in the ASFV TAR fragments. The individual fragments were modified by cleavage *in vitro* by Cas9 nuclease guided by target gene-specific gRNAs and then co-transformed with a "fix" containing the fluorescent gene flanked by 45 bp of homology to either side of the targeted gene locus into yeast cells. Yeast transformants were screened by PCR amplification and positive clones were then transferred to *E. coli*. *E. coli* transformants were also screened by PCR amplification and restriction enzyme digestion. **A.** A representative agarose gel after PCR amplification showing the presence of the modified O61R gene (immune from Cas6 cleavage) using primers, ASFV O61R Change 5' Det and ASFV O61R Change 3' Det as well as the 5' and 3' junctions of ASFV Fragment 9 in yeast and *E. coli*. **B.** A representative agarose gel after PCR amplification showing the replacement of the K145R gene with mCherry at the 5' junction and presence of the 3' junction of ASFV Fragment 5 in yeast and *E. coli*. **C.** A representative agarose gel after PCR amplification showing the deletion of the A238L gene as well as the 5' and 3' junctions of ASFV Fragment 4 in yeast and *E. coli*. **D.** A representative agarose gel after PCR amplification showing the deletion of the I329L gene as well as the 5' and 3' junctions of ASFV Fragment 12 in yeast and *E. coli*. **E.** A representative agarose gel after PCR amplification showing the fusion of mCherry to the CD2v gene as well as the 5' and 3' junctions of ASFV Fragment 5 in yeast and *E. coli*. **F.** A representative agarose gel after PCR amplification showing the fusion of Cerulean to the K145R gene as well as the 5' and 3' junctions of ASFV Fragment 5 in yeast and *E. coli*.

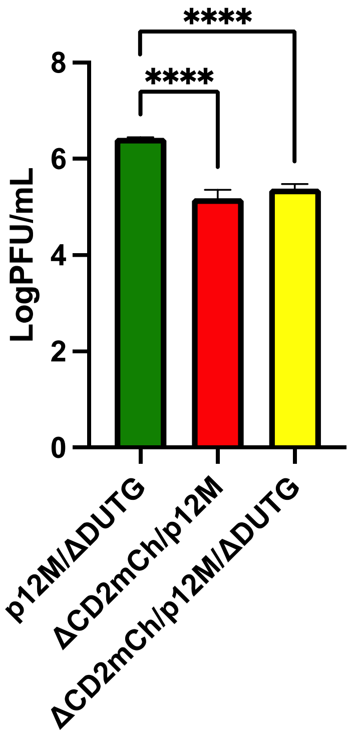

Fig. S6. Viral titers of progeny after reconstitution of ΔCD2vmCherry using self-helper virus ΔDUT::GFP. The ΔCD2vmCherry/p12M virus was reconstituted by transfecting the corresponding full-length genome into WSL cells and then infecting with the ΔDUT::GFP virus. Analysis of the virus progenies from two independent experiments by plaques assays on WSL cells revealed green (ΔDUTG), red (ΔCD2vmCherry/p12M), double-fluorescent (ΔCD2vmCherry/p12M/ΔDUTG) and nonfluorescent (p12M, not counted) virus recombinants. Shown are the proportionate titers of the different fluorescent recombinants with positive standard deviation.

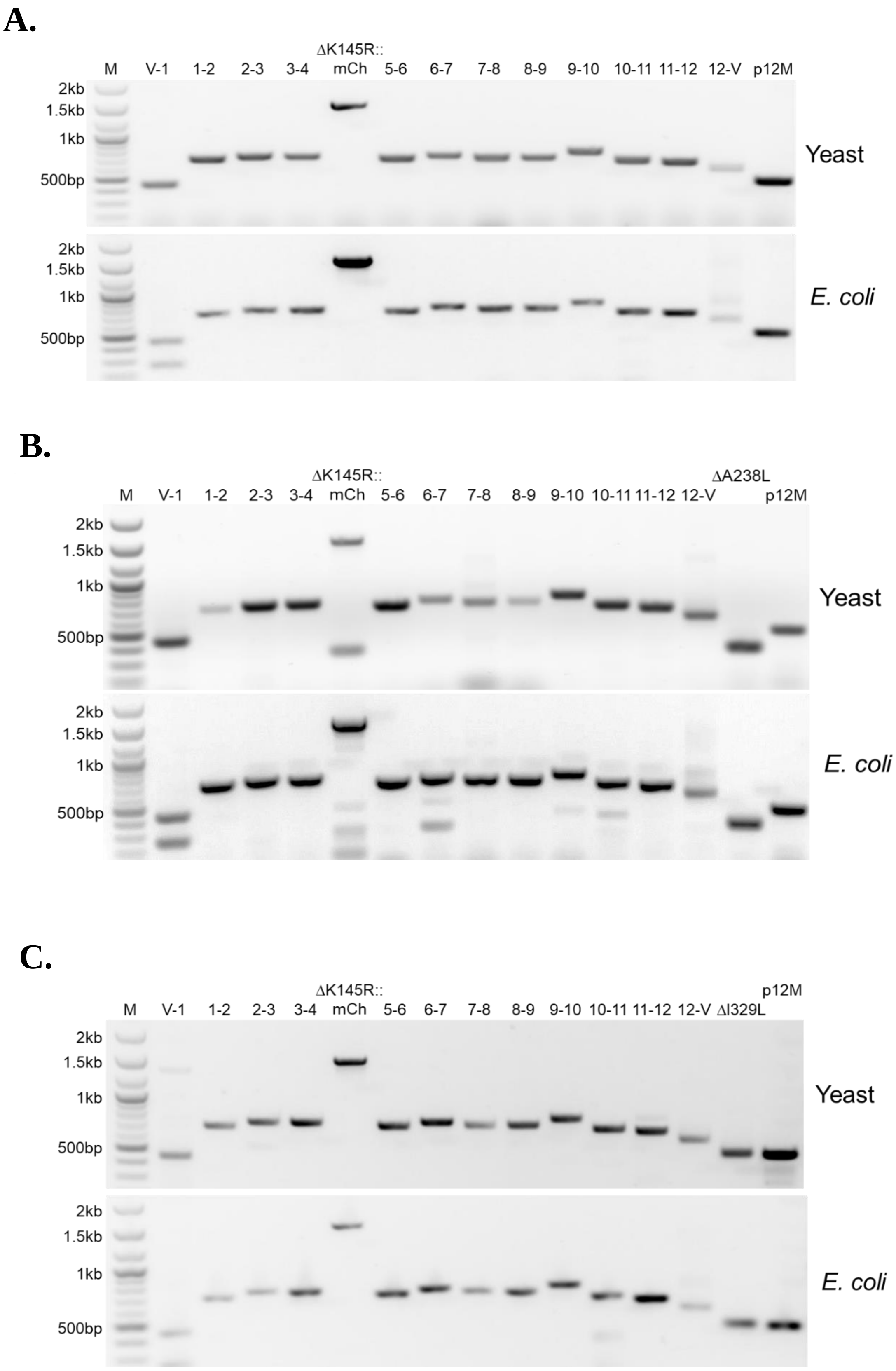
A.
M
V-1
1-2
2-3
3-4
ΔK145R::
mCh
5-6
6-7
7-8
8-9
9-10
10-11
11-12
12-V
p12M
2kb
1.5kb
1kb
500bp
Yeast
E. coli
B.
M
V-1
1-2
2-3
3-4
ΔK145R::
mCh
5-6
6-7
7-8
8-9
9-10
10-11
11-12
12-V
ΔA238L
p12M
2kb
1.5kb
1kb
500bp
Yeast
E. coli
C.
M
V-1
1-2
2-3
3-4
ΔK145R::
mCh
5-6
6-7
7-8
8-9
9-10
10-11
11-12
12-V
p12M
ΔI329L
2kb
1.5kb
1kb
500bp
Yeast
E. coli

D.

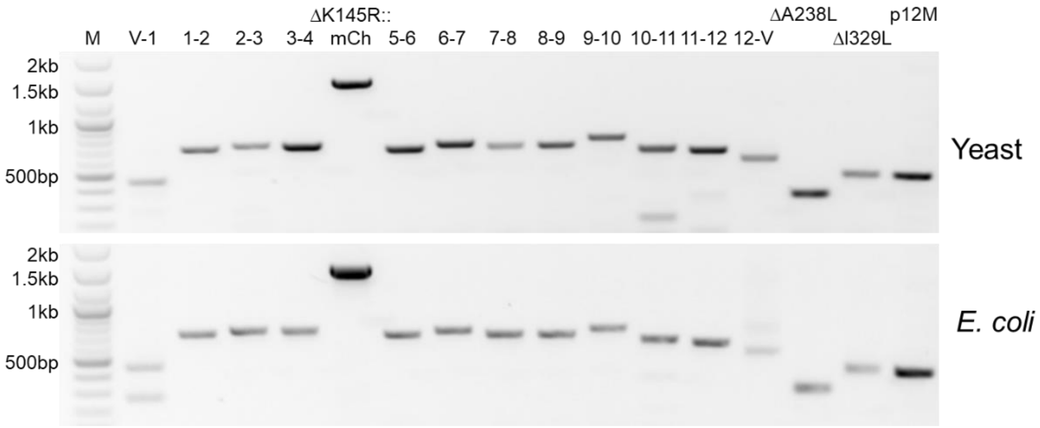
M
V-1
1-2
2-3
3-4
ΔK145R::
mCh
5-6
6-7
7-8
8-9
9-10
10-11
11-12
12-V
ΔA238L
ΔI329L
p12M
2kb
1.5kb
1kb
500bp
Yeast
2kb
1.5kb
1kb
500bp
E. coli

E.

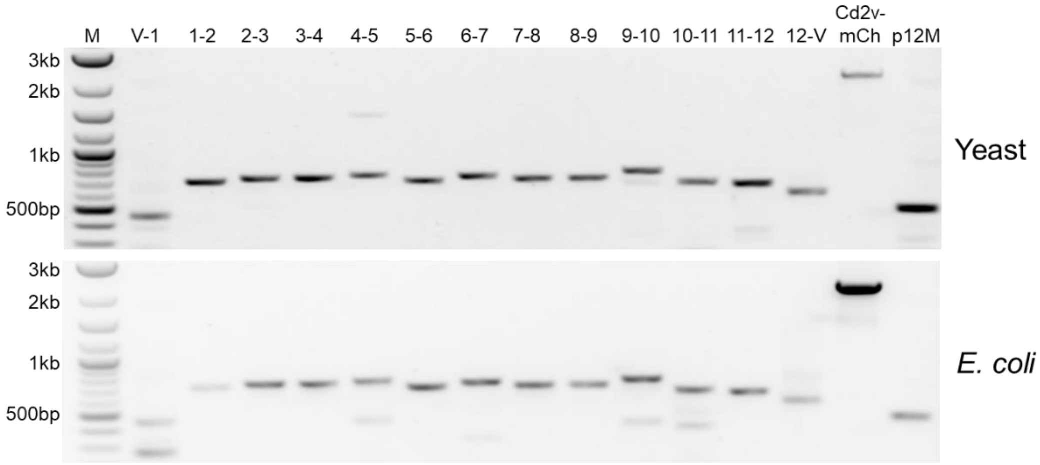
M
V-1
1-2
2-3
3-4
4-5
5-6
6-7
7-8
8-9
9-10
10-11
11-12
12-V
Cd2v-
mCh
p12M
3kb
2kb
1kb
500bp
Yeast
3kb
2kb
1kb
500bp
E. coli

F.

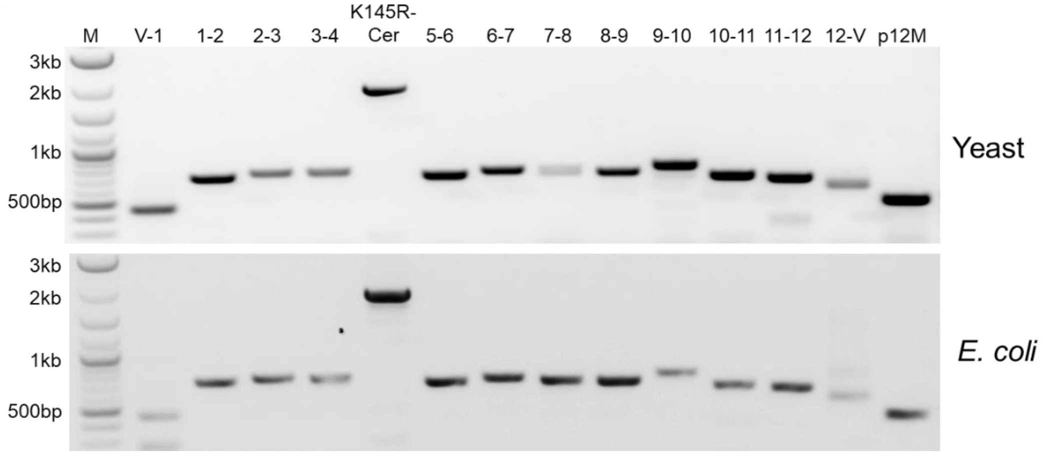
M
V-1
1-2
2-3
3-4
K145R-
Cer
5-6
6-7
7-8
8-9
9-10
10-11
11-12
12-V
p12M
3kb
2kb
1kb
500bp
Yeast
3kb
2kb
1kb
500bp
E. coli

Fig. S7. Assembly of ASFV full-length genomes containing replacements, deletions and modifications of various genes. Assembly of modified ASFV full-length genomes was performed by first assembling one-third genomes from a mix of appropriate I-SceI-cleaved wild-type and modified fragments by TAR in yeast followed by transfer of positive clones into *E. coli*. Then, full-length genomes were assembled from the appropriate I-SceI-cleaved one-third genomes by TAR in yeast followed again by transfer to *E. coli*. The assemblies were screened by junction PCR amplification in both yeast and *E. coli*. **A.** A representative agarose gel after PCR amplification showing the presence of all appropriate junctions in the genome containing the replacement of K145R with mCherry in the modified P12 background. The K145R replacement junction was amplified using Det_04_3p and ASFV K145R Del 3' Det as primers. **B.** A representative agarose gel after PCR amplification showing the presence of all appropriate junctions in the genome containing the replacement of K145R with mCherry and deletion of A238L in the modified P12 background. In addition, the deletion of A238L was confirmed by PCR amplification using primers, ASFV A238L Del 5' Det and ASFV A238L Del 3' Det. **C.** A representative agarose gel after PCR amplification showing the presence of all appropriate junctions in the genome containing the replacement of K145R with mCherry and deletion of I329L in the modified P12 background. In addition, the deletion of I329L was confirmed by PCR amplification using primers, ASFV I329L Del 5' Det and ASFV I329L Del 3' Det. **D.** A representative agarose gel after PCR amplification showing the presence of all appropriate junctions in the genome containing the replacement of K145R with mCherry as well as deletions of A238L and I329L in the modified P12 background. In addition, the deletions of A238L and I329L were confirmed by PCR amplification as above. **E.** A representative agarose gel after PCR amplification showing the presence of all appropriate junctions in the genome containing the fusion of CD2v with mCherry in the modified P12 background. In addition, the CD2v-mCherry fusion was confirmed by PCR amplification using primers, ASFV CD2v Del 5' Det and ASFV CD2v Del 3' Det. **F.** A representative agarose gel after PCR amplification showing the presence of all appropriate junctions in the genome containing the fusion of K145R with Cerulean in the modified P12 background. In addition, the K145R-Cerulean fusion was confirmed by PCR amplification. The K145R-Cerulean fusion junction was amplified using Det_04_3p and ASFV K145R Del 3' Det as primers. For all of the genomes, the P12 modification was confirmed by PCR amplification using primers, ASFV O61R Change 5' Det and ASFV O61R Change 3' Det.

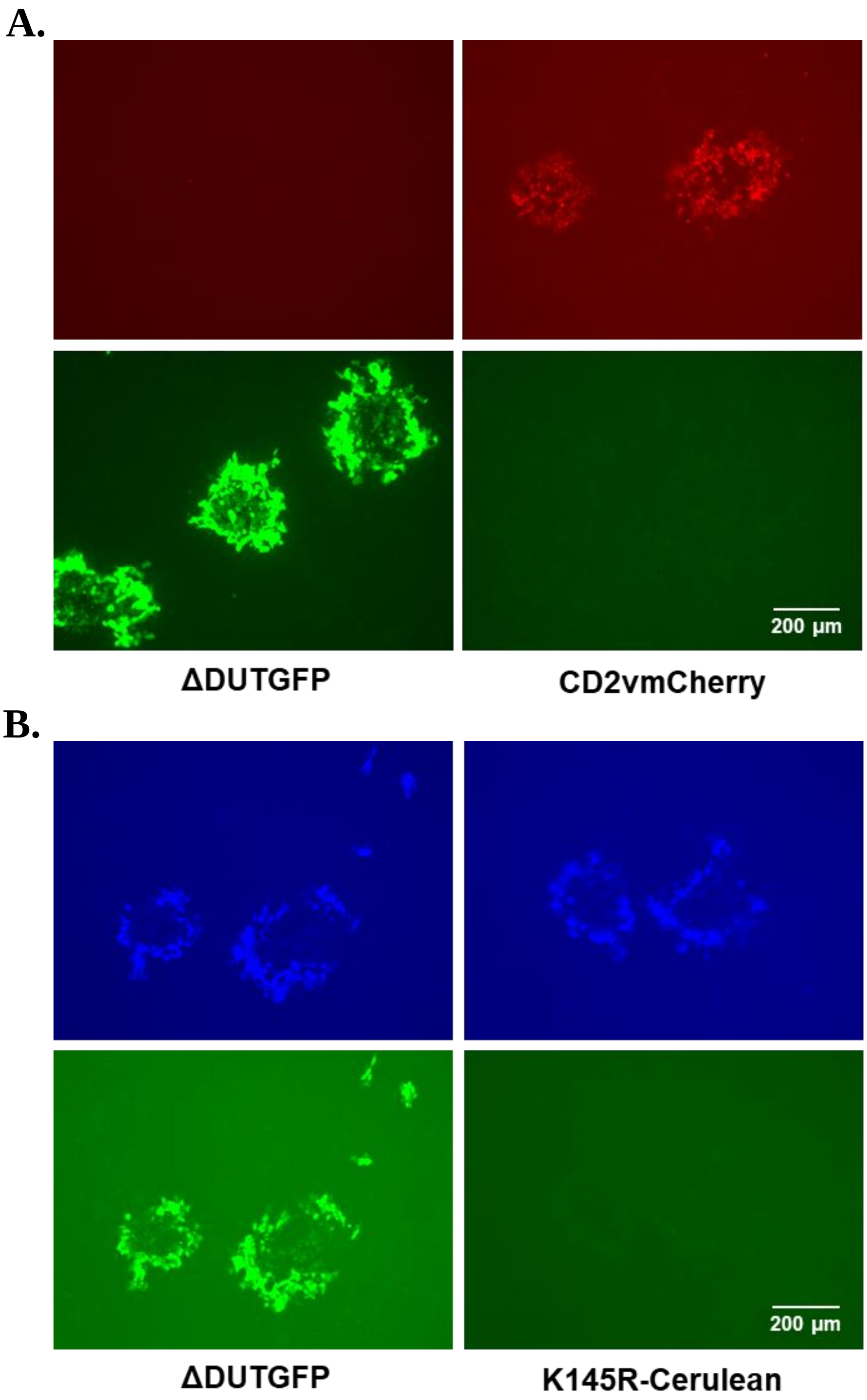

Figure S8. Multichanel images of representative virus plaques (6 d p.i.) of ASFV recombinants expressing fluorescent fusion proteins. A. ASFV recombinant containing a CD2v-mCherry fusion protein (**A**) or a K145R-Cerulean fusion protein (**B**) compared to plaques of the parental helper virus ASFV-Kenya ΔDUTGFP. Note that GFP fluorescence is also excited by the cerulean blue filter (CFP Ex 436/20).

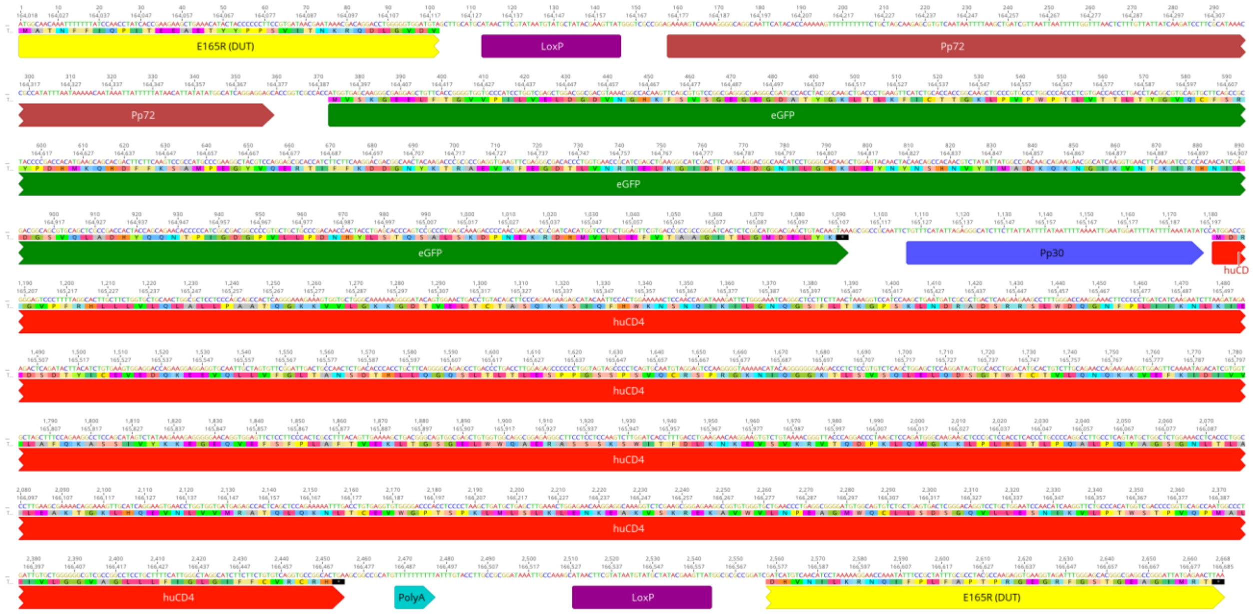

Figure S9. Map with nucleotide and amino acid sequences of the mutated genome region of ASFV-Kenya ΔDUTGFP. The viral dUTPase-encoding ORF E165R was partly deleted, and substituted by genes encoding marker proteins (eGFP, human CD4) under control of strong ASFV promoters (Pp72, Pp30). An ASFV-specific polyadenylation signal and flanking LoxP sites permitting subsequent removal of the reporter gene cassette by Cre recombinase are also indicated.

No.	Cells	Helper virus	TR	A104R	K196R (TK)	CP204L (p30)	E199L
			~ 500	~ 43,000	~ 61,000	~ 122,000	~ 164,000
1 (P11)	WSL-HP	NHVΔTKG	Kenya	Kenya	Kenya	Kenya	Kenya
2 (P21)	WSL-HP	NHVΔTKG	Kenya	Kenya	Kenya	NHV	Kenya
3 (P11)	WSL-HP	ArmΔ285LG	Kenya	Kenya	Kenya	Kenya	Kenya
4 (P21)	WSL-HP	ArmΔ285LG	Kenya	Kenya	Kenya	Kenya	Kenya
5 (P11)	WSL-HP	Armenia lp	Kenya	Kenya	Kenya	Kenya	Kenya
6 (P21)	WSL-HP	Armenia lp	Kenya	Kenya	Kenya	Kenya	Kenya
7 (P11)	WSL-gRCP204L	NHVΔTKG	Kenya	Kenya	Kenya	Kenya	Kenya
8 (P21)	WSL-gRCP204L	NHVΔTKG	Kenya	Kenya	Kenya	Kenya	Kenya
9 (P11)	WSL-gRCP204L	ArmΔ285LG	Kenya	Kenya	Kenya	Kenya	Kenya
10 (P21)	WSL-gRCP204L	ArmΔ285LG	Kenya	Kenya	Kenya	Kenya	Kenya

Table S1. Booting up of recombinant DNA of ASFV KenyaΔCD2DsRed on transfected WSL or CRISPR/Cas9-expressing (WSL-gRCP204L) cells using different ASFV helper viruses. Designations, genome positions and origins of analyzed progeny virus genes are indicated.

**ASFV Kenya IX 1033 (GenBank # OZ005801)**		**ΔCD2v::DsRed (rescued from genomic DNA)**		**ΔCD2v::mCh (heterologous helper virus)**		**ΔCD2v::mCh/p12M (homologous helper virus)**	
**position**	**gene**	**mutation**	**effect**	**mutation**	**effect**	**mutation**	**Effect**
2150	-	+ TT	?	+ TT	?	+ TT	?
2194	-	A → C	?	A → C	?	A → C	?
2435	-	HP - T	?				
13008	MGF 110-11L	HP + C	frameshift repair	HP + C	frameshift repair	HP - C	frameshift change
15201	-			HP + GGG	?		
17415	-					HP + GGG	?
17617	-					HP + GG	?
20158	MGF 300-2R	HP - A	frameshift	HP - A	frameshift	HP - A	frameshift
32307	MGF 505-2R					G → A	Ala → Thr
70324 - 71442	EP402R (CD2v)			substitution	ΔCD2vmCherry	substitution	ΔCD2vmCherry
70557 - 71442	EP402R (CD2v)	substitution	ΔCD2vDsRed				
96866	-			A → G	?		
126331	O61R					A → G	silent, Cas9 resist.
126334	O61R					T → C	silent, Cas9 resist.
126337	O61R					T → C	silent, Cas9 resist.
126340	O61R					T → A	silent, Cas9 resist.
126343	O61R					A → T	silent, Cas9 resist.
126346	O61R					T → C	silent, Cas9 resist.
135367	D250R	G → A	Ala → Thr			G → A	Ala → Thr
162317	E301R	T → C	silent				
162332	E301R	T → C	silent				

Table S2. Reconstituted ASFV isolates are identical to parental strain except for the desired modification. Yellow: "mutations" found in all hitherto analyzed ASFV Kenya IX 1033 recombinants, possibly due to errors in the original sequence. **Green:** desired mutations. **Blue:** mutation in an intergenic region only found in surely BAC derived mutants, possibly occurred during cloning. **Red:** unwanted mutations leading to altered amino acid sequences of viral proteins. Alterations in homopolymer (HP) length can frequently arise during virus or plasmid replication and may also partly be due to sequencing errors.

Fragment	Size (bp)	⅓ Genomes (bp)
1	12116	61860
2	19304	
3	14508	
4	15932	
5	13704	59329
6	15095	
7	15486	
8	15044	
9	15095	66735
10	14797	
11	15130	
12	21713	

Table S3. ASFV TAR Fragment and one third genome sizes.

	Name	Size (bases)	Sequence
Fragment 1	Con_01_5p	83	TGCACTATAATATAAAATTGTACCTGCTTTATATATAATAAATTTTAGGGATAACAGGGTAATCGGGTACCGAGCTCGAATTC
	Con_01_3p	83	CTGTATGTAATCATTTTTTTTGATGAGAGGATGTTTTTCAAAAACTAGGGATAACAGGGTAATGATCCTCTAGAGTCGACCTG
Fragment 2	Con_02_5p	83	TTACATACAGATATGTAGGGTTTCCCGCTATGGTGAAAAATGTAGTAGGGATAACAGGGTAATCGGGTACCGAGCTCGAATTC
	Con_02_3p	83	TCGCTCTTCACGGCCTTTACTAGCGCCGCGTCAAGACAAAGATCATAGGGATAACAGGGTAATGATCCTCTAGAGTCGACCTG
Fragment 3	Con_03_5p	83	TGAAGAGCGATAATCTTGAGCTTATACGATTGTTTGTGGATTGGGTAGGGATAACAGGGTAATCGGGTACCGAGCTCGAATTC
	Con_03_3p	83	AAAGCTTATAGAATGCATTGATAATGAGCTGCAAAACAGTGGAACTAGGGATAACAGGGTAATGATCCTCTAGAGTCGACCTG
Fragment 4	Con_04_5p	83	TATAAGCTTTTCTTTGTGTAACAACGCCATTATAACAATTATTTTTAGGGATAACAGGGTAATCGGGTACCGAGCTCGAATTC
	Con_04_3p	83	CACCTGTATGAAATGTAACCGACATAATGCATGCTTTAATGTGCGTAGGGATAACAGGGTAATGATCCTCTAGAGTCGACCTG
Fragment 5	Con_05_5p	83	CATACAGGTGCGACCAATATACTTAACCCAGTTGCAGTAAGGAAATAGGGATAACAGGGTAATCGGGTACCGAGCTCGAATTC
	Con_05_3p	83	ACGCTTCTGCCGCTTGAAGCTGTATAAGCATGTCCACATGAGGACTAGGGATAACAGGGTAATGATCCTCTAGAGTCGACCTG
Fragment 6	Con_06_5p	83	GCAGAAGCGTCCTACGACACCATGCGAACCAAGCTAATGAAATTTTAGGGATAACAGGGTAATCGGGTACCGAGCTCGAATTC
	Con_06_3p	83	TTTGTTCGTCGTAGCAGAAACAATTAAGGCGATACGCCTCATAAATAGGGATAACAGGGTAATGATCCTCTAGAGTCGACCTG
Fragment 7	Con_07_5p	83	GACGAACAAAGGCTTCTTTATACGGGAAGAAATGGAATACACTTTTAGGGATAACAGGGTAATCGGGTACCGAGCTCGAATTC
	Con_07_3p	83	ATTAAGCTATTGCAAATCGCCGAGCCGGTGAAGGCGTTGAACTGCTAGGGATAACAGGGTAATGATCCTCTAGAGTCGACCTG
Fragment 8	Con_08_5p	83	ATAGCTTAATTAACTCCATATGCGAACATGCAAGGCTGCTAATTTTAGGGATAACAGGGTAATCGGGTACCGAGCTCGAATTC
	Con_08_3p	83	CGCGGCCTTGCTGAGCCCGAGCTCCTCCACCGTTAGGGCAGCACCTAGGGATAACAGGGTAATGATCCTCTAGAGTCGACCTG
Fragment 9	Con_09_5p	83	CAAGGCCGCGCGGTCGCAGGTTGACCTCAACCAGGCAATTAACACTAGGGATAACAGGGTAATCGGGTACCGAGCTCGAATTC
	Con_09_3p	83	TTATGTAAGTCTTTGGAGGAACCCAATATGAATCTTTCTTTACAATAGGGATAACAGGGTAATGATCCTCTAGAGTCGACCTG
Fragment 10	Con_10_5p	83	ACTTACATAACACTGCAAGGAAGTATATATGCTTATATTCTGTTTTAGGGATAACAGGGTAATCGGGTACCGAGCTCGAATTC
	Con_10_3p	83	TCTTGCTTTAGGCGATGAAGACAAAGGTATGGACAGCATGTTAAATAGGGATAACAGGGTAATGATCCTCTAGAGTCGACCTG
Fragment 11	Con_11_5p	83	TAAAGCAAGAATATGTTGATAATAATCGCTCAGTTCAGTATTTACTAGGGATAACAGGGTAATCGGGTACCGAGCTCGAATTC
	Con_11_3p	83	TCCTATCCAGTATTTGAAAGATGATTTCAAGGACCAAACCTCTATTAGGGATAACAGGGTAATGATCCTCTAGAGTCGACCTG
Fragment 12	Con_12_5p	83	CTGGATAGGAGAATTAAGATCTGCCATCACTATAACAATACCTTTTAGGGATAACAGGGTAATCGGGTACCGAGCTCGAATTC
	Con_12_3p	83	TGCACTATAATATAAAATTGTACCTGCTTTATATATAATAAATTTTAGGGATAACAGGGTAATGATCCTCTAGAGTCGACCTG

Table S4. Construction primers to clone the ASFV-Kenya1033 fragments. Each primer contains an ASFV homology "hook" (black text), an I-SceI recognition sequence (red text) and YCpBAC sequence (blue text) to amplify a YCpBAC vector that then contains ASFV homology "hooks" to clone each targeted ASFV fragment flanked by I-SceI recognition sequences.

	Name	Sequence	Size
Fragment 1	Det_01_5p	ATGTTTAACGCCAACAGCAGC	446 bp
	Det_01_3p	GAGGCAGGTTCATTATGATG	481 bp
Fragment 2	Det_02_5p	AACAGATACATTACCAAGAT	478 bp
	Det_02_3p	CTTCGTTAATAGGAGGTGCG	446 bp
Fragment 3	Det_03_5p	GAGGAATGAAGGCTTTGCAAG	513 bp
	Det_03_3p	GCAATAAGTACTCCACGCATG	505 bp
Fragment 4	Det_04_5p	GGACCAACTGAGGTTGAAAC	454 bp
	Det_04_3p	GGAACAATTAGCCTTGTGCTG	534 bp
Fragment 5	Det_05_5p	CAGCTGTTGTATGCTGCGGTA	444 bp
	Det_05_3p	CGGCATACAGCTGAGTTAAAG	484 bp
Fragment 6	Det_06_5p	CAAGGTGGATGACCCTAGCAG	435 bp
	Det_06_3p	GGAAACGTAATCGTCTGATG	491 bp
Fragment 7	Det_07_5p	CAATGCTTATAGCAACATCC	471 bp
	Det_07_3p	GGCACGAATTTCGGGACAGT	517 bp
Fragment 8	Det_08_5p	TGCAAGAATTCTACCCACTG	420 bp
	Det_08_3p	AAGGTAAAGGTCCAGCCCTT	475 bp
Fragment 9	Det_09_5p	CCGAACATAACGAAGCTTCG	454 bp
	Det_09_3p	CGCATGTGGTATCATATTTGG	460 bp
Fragment 10	Det_10_5p	ATAGAGGAGGATGACGAGTA	533 bp
	Det_10_3p	CTTGCGGCGTTTAACCAGCAG	468 bp
Fragment 11	Det_11_5p	GGTACGGACCTATCATACTC	433 bp
	Det_11_3p	CAACTCAGAAGCAGTCGCAG	505 bp
Fragment 12	Det_12_5p	TCCGGTTTATTGTCCGCCTC	379 bp
	Det_12_3p	GCCTACCACAGCAGTAGGAA	567 bp
	RCO495	ACGACGGCCAGTGAATTG	
	RCO493	CCAAGCTATTTAGGTGAGAC	

Table S5. Detection primers to confirm TAR cloned ASFV fragments and assembled genomes. For TAR-cloned fragments, the 5p primers were used with RCO495 to amplify the junction between the 5’ end of the fragment and the vector and the 3p primer were used with RCO493 to amplify the junction between the 3’ end of the fragment and the vector. For TAR-assembled genomes, the 5p primer was used with the adjacent fragment 3p primer to amplify the junction between each appropriate fragment.

Name	Sequence
ASFV BA71V Slow	ATATATATAAAATTATAAAATATATAATATACTTATA**TTAT**
ASFV BA71V Fast	TATAAGTATATTATATATTTTATAATTTTATATATAT**TTAT**
ASFV Kenya Slow	ATATATAATAAATTTATAATTTATATAATAAATTTATA**TTAT**
ASFV Kenya Fast	TATAAATTTATTATATAAATTATAAATTTATTATATAT**TTAT**
ASFV Kenya*	TTTCACAAAAAAGATCTGCACTATAATATAAAATTGTACCT GCTTTTATAAATTTATTATATAAA**TTAT**

Table S6. Hairpin loop sequences ligated to assembled ASFV genomes. The sequence in red is complementary to the overhang sequence remaining after cleaving assembled ASFV genomes with I-SceI. ***Present in ΔCD2v::mCh reconstituted with heterologous helper virus.

ASFV Kenya IX 1033 (GenBank # OZ005801)		ΔA238LΔK145RmChΔI329L/p12M (YCpBAC)		ΔA238LΔK145RmChΔI329L/p12M (virus)		ΔDUT-LoxP/GFP/CD4 (self-helper virus)	
**position**	**gene**	**mutation**	**effect**	**mutation**	**effect**	**mutation**	**Effect**
2150	-	+ TT	?	+ TT	?	+ TT	?
2194	-	A → C	?	A → C	?	A → C	?
13008	MGF 110-11L	HP + C	frameshift repair			HP - C	frameshift change
15201	-	HP + GGG	?				
17415	-			HP + GGG	?	HP + GGG	?
17617	-			HP + GG	?	HP + GG	?
20158	MGF 300-2R	HP - A	frameshift	HP - A	frameshift	HP - A	Frameshift
26250	-			T → C	?		
32307	MGF 505-2R			G → A	Ala → Thr	G → A	Ala → Thr
47421 - 48140	A238L	deletion	ΔA238L	deletion	ΔA238L		
61723 - 62160	K145R	replacement	ΔK145RmCherry	replacement	ΔK145RmCherry		
96866	-	A → G	?				
126331	O61R	A → G	silent, Cas9 resist.	A → G	silent, Cas9 resist.		
126334	O61R	T → C	silent, Cas9 resist.	T → C	silent, Cas9 resist.		
126337	O61R	T → C	silent, Cas9 resist.	T → C	silent, Cas9 resist.		
126340	O61R	T → A	silent, Cas9 resist.	T → A	silent, Cas9 resist.		
126343	O61R	A → T	silent, Cas9 resist.	A → T	silent, Cas9 resist.		
126346	O61R	T → C	silent, Cas9 resist.	T → C	silent, Cas9 resist.		
135367	D250R			G → A	Ala → Thr	G → A	Ala → Thr
164115 - 164399	E165R					substitution	ΔDUT-LoxP/GFP/CD4
170186 - 171175	I329L	deletion	ΔI329L	deletion	ΔI329L		

Table S7. Reconstituted triple mutant ASFV is identical to parental strain except for the desired modifications. Yellow: "mutations" found in all hitherto analyzed ASFV Kenya IX 1033 recombinants, possibly due to errors in the original sequence. **Green:** desired mutations. **Blue:** mutation in an intergenic region only found in surely BAC derived mutants, possibly occurred during cloning. **Red:** unwanted mutations leading to altered amino acid sequences of viral proteins. Alterations in homopolymer (HP) length can frequently arise during virus or plasmid replication and may also partly be due to sequencing errors.

Name	Sequence	Description
CRISPR ultramer	GTTTTAGAGCTAGAAATAGCAAGTTAAAATAAGGCTAGTCCGTTATCAACTTGAAAAAGTGGCACCGAGTCGGTGCTTTTTTT	Oligo template for amlification of CRISPR sgRNA DNA template
CRISPR R	AAAAAAAGCACCGACTCG	Reverse primer to amplify CRISPR template
CRISPR F gRNA1 CD2v	TAATACGACTCACTATAGGAATTTACTTACCACATGTGTTTTAGAGCTAGAAATAGCAA	gRNA at 5' end of ASFV CD2v
CRISPR F gRNA2 CD2v	TAATACGACTCACTATAGGAGGATATGGTTCAGGTGAGTTTTAGAGCTAGAAATAGCAA	gRNA at 3' end of ASFV CD2v
ASFV CD2v Flu Rep 5' Fix	ATAGTTTTAAAAATATTATTATAAAATATATGAGTATAAATCCTTCGCATAAACCGCCATATTTAATAAAAACAATAAATTATTTTTATAACATTATAT	Oligo with homology to 5' region of the CD2 locus and p72 promoter to express fluorecent gene
ASFV Flu Rep 3' Fix 5'	TATTTAATAAAAACAATAAATTATTTTTATAACATTATATATGGTGAGCAAGGGCGAGGA	Primer to amplify mCherry gene to replace any ASFV gene
ASFV CD2v Flu Rep 3' Fix 3'	ATTAAATAATTCTATCTACGTGAATAAGCGAAATATTTTGCTACTTGTACAGCTCGTCCA	Primer to amplify mCherry gene to replace CD2v
ASFV Cd2v mCh Fusion 5’	TACACAAAATATTTCGCTTATTCACGTAGATAGAATTATTATGGTGAGCAAGGGCGAGGAG	Primer to amplify mCherry gene for fusion to CD2v
ASFV Cd2v mCh Fusion 3’	ATAGTACATATGGTACATACGGTACATATGGTACATATTACTTGTACAGCTCGTCCAT	Primer to amplify mCherry gene for fusion to CD2v
ASFV CD2v mCh Fusion Fix 5'	CTAATAATCCTACATTAAATG	Primer to amplify CD2v for mCherry fusion
ASFV CD2v mCh Fusion Fix 3'	AATAATTCTATCTACGTGAATAAG	Primer to amplify CD2v for mCherry fusion
ASFV CD2v Del 5' Det	GTCATTAGCATTACAGAATAG	Primer to confirm replacement of CD2v and fusion to mCherry
ASFV CD2v Del 3' Det	CACATGATGCTCTCGATGATC	Primer to confirm replacement of CD2v and fusion to mCherry
CRISPR F gRNA1 K145R	TAATACGACTCACTATAGGGTATAAGCTAGATCCCATGTTTTAGAGCTAGAAATAGCAA	gRNA at 5' end of ASFV K145R
CRISPR F gRNA2 K145R	TAATACGACTCACTATAGGATATTTATAAGAAGCTTGGTTTTAGAGCTAGAAATAGCAA	gRNA at 3' end of ASFV K145R

ASFV K145R Flu Rep 5’ Fix	ATGGTTGCGACCTATTAAATAATAAATATATTATACTATATCCTTCGCATAAACCGCCATATTTAATAAAAACAATAAATTATTTTTATAACATTATAT	Oligo with homology to 5' region of the K145R locus and p72 promoter to express fluorecent gene
ASFV K145R Flu Rep 3' Fix 3'	CATATTCCTATAAAAATAGCAAAAAAAAATGTAAGTTGTCCTACTTGTACAGCTCGTCCAT	Primer to amplify mCherry gene to replace K145R
ASFV K145R Cerulean Fusion 5'	AACCTGGGCAAAGATTGTTGAAGAAGGAGGAGAAGAATCCGTGAGCAAGGGCGAGGAGCT	Primer to amplify Cerulean gene for fusion to K145R
ASFV K145R Cerulean Fusion 3'	ATTCCTATAAAAATAGCAAAAAAAAATGTAAGTTGTCTTACTTGTACAGCTCGTCCA	Primer to amplify Cerulean gene for fusion to K145R
ASFV K145R Cerulean Fusion Fix 5'	TATTATACTATAATGGATC	Primer to amplify K145R for Cerulean fusion
ASFV K145R Cerulean Fusion Fix 3'	GGATTCTTCTCCTCCTTCTTC	Primer to amplify K145R for Cerulean fusion
ASFV K145R Del 3’ Det	TAGCACGTCCAAGTAGCGATC	Primer to confirm replacement of K145R and fusion to Cerulean
CRISPR F gRNA A238L	TAATACGACTCACTATAGGGAACACTTGTTTATTAACGTTTTAGAGCTAGAAATAGCAA	gRNA at 5' end of ASFV A238L
ASFV A238L Del Fix	TAATACAGAGAATTTAGATCATTAAGATCATCAAGAGATATTTTAGGTAGTCCTCGTACTCTCACTTTATAAGGAGTATT	Oligo with homology to either end of the ASFV A238L gene to delete it.
ASFV A238L Del 5’ Det	TCTTACGCCATGCGCGACTAA	Primer to confirm A238L deletion
ASFV A238L Del 3’ Det	CGGCTTATCCAAAGAAGTCCG	Primer to confirm A238L deletion
CRISPR F gRNA1 I329L	TAATACGACTCACTATAGGTAATGGATACCAACACAAGTTTTAGAGCTAGAAATAGCAA	gRNA at 3' end of ASFV I329L
CRISPR F gRNA2 I329L	TAATACGACTCACTATAGGTATACCAACTAATGCTACGTTTTAGAGCTAGAAATAGCAA	gRNA at 5' end of ASFV I329L
ASFV I329L Del Fix	GAAATGGCAGATATACACATGTTAAACATAAGCCACATATCCTGTATATACTATTAAAAATTTATAAAATTAATTTTGTT	Oligo with homology to either end of the ASFV I329L gene to delete it.
ASFV I329L Del 5’ Det	GGTATATAGGAGATTATAGGT	Primer to confirm I329L deletion
ASFV I329L Del 3’ Det	CCATACTTTTAGTTGCATAAC	Primer to confirm I329L deletion

CRISPR F gRNA O61R	TAATACGACTCACTATAGGACTTGATGGTTCAAGTGGGTTTTAGAGCTAGAAATAGCAA	gRNA for ASFV O61R
ASFV O61R Change Fix	TAAAATCTTTTCATTTTATATATTATATACGCAAAATGGCGCTCGACGGATCTAGCGGTGGAGGCTCTAATGTAGAAACATTACTTATTGTAGCAA	Oligo with modifications to gRNA recognition sequence and homology to either side of the modifications in ASFV O61R to immunize it against cleavage by Cas9.
ASFV O61R Change 5' Det	ATGGCGCTCGACGGATCTAGC	Primer to confirm modifications to O61R
ASFV O61R Change 3' Det	CTTGACGGATGCTGCGATTG	Primer to confirm modifications to O61R

Table S8. Primer sequences used to modify genes and confirm the modifications in the ASFV TAR fragments.

Primer	Sequence	Genome position in ASFV-Kenya-IX-1033
AKT-PSF	5'-AGATGCAGAATTTCGCGG-3'	135 – 152<br>186,864 – 186,881 (R)
AKT-PSR	5'-GGTTGCCCACTAGTATGC-3'	757 – 774 (R)<br>186,242 – 186,259
AKA104RRHF-F	*5'-GAATTCGAGCTCGGTACCC*AACCAAG GAAGAAATTAGCG-3'	43,083 – 43,102
AKA104RRHF-R	*5'-GTCGACTCTAGAGGATCCC*CAATGTT GTTGGATGTTGCTC-3'	45.856 – 45,876 (R)
ASFVKTK-F	5'-ATCTAAGGGCATTCCGATTTAC-3'	61,038 – 61,059
ASFVKTK-R	5'-TTTATTATTTAATAGGTCGCAACC-3'	61,685 – 61,708 (R)
CP204L-PSF	5'-CACAAGTTGTGTTTCATGC-3'	122,805 – 122,823 (R)
CP204L-PSR	5'-GGGTAACCGTGGATCTTCA-3'	122,143 – 122,161
ASFVKE199L-F	5'-TTCAGCTTCTTCGGTGATAGGTTGG-3'	164,033 – 164,057 (R)
ASFVKE199L-R	5'-AAGTCTTGCTCATGGCTTACGTTAC-3'	163,253 – 163,277

Table S9. Primers used to amplify and sequence relevant regions in the ASFV genome. 5' extensions used for cloning are in italics.

Supplementary Data (separate files)

Data S1: Sequence of ASFV Kenya ΔCD2v::DsRed (D).

Annotated genome sequence of the reconstituted ΔCD2v::DsRed virus from transfected virion DNA.

Data S2: Annotated sequence file of ASFV Kenya ΔCD2v::mCh.

Annotated genome sequence of the reconstituted ΔCD2v::mCh virus from transfected "synthetic" assembled genome.

Data S3: Annotated sequence file of ASFV Kenya ΔCD2v::mCh/p12M.

Annotated genome sequence of the reconstituted ΔCD2v::mCh/p12M virus from transfected "synthetic" assembled genome.

Data S4: Annotated sequence file of ASFV Kenya ΔK145R::mCh/ΔA238L/ΔI329L/p12M.

Annotated genome sequence of the reconstituted ΔK145R::mCh/ΔA238L/ΔI329L/p12M virus from transfected "synthetic" assembled genome.

Data S5: Annotated sequence file of ASFV Kenya ΔDUT::GFP.

Annotated genome sequence of the helper virus ΔDUT::GFP.
